# A Compact Chemical Kinetic Mechanism for Heavy Fuel Surrogates with *n*-, *iso*- and *cyclo*-Alkanes, and Aromatic Compounds

**DOI:** 10.1021/acsomega.5c00158

**Published:** 2025-04-11

**Authors:** Niklas Zettervall, Elna J. K. Nilsson

**Affiliations:** † Weapons, Protection and Security, Swedish Defence Research Agency FOI, 164 90 Stockholm, Sweden; ‡ Division of Combustion Physics, Lund University, Box 118, 22100 Lund, Sweden

## Abstract

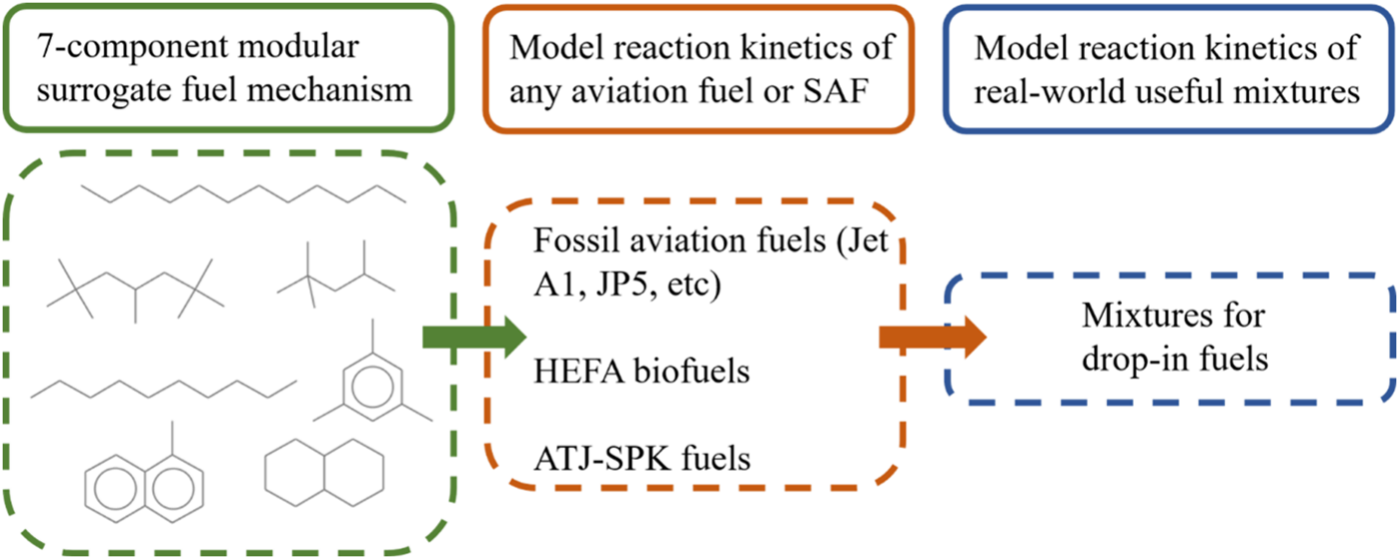

Modeling of real-world fuels and fuel mixtures in combustion engines, using computational fluid dynamics (CFD), calls for the development of compact and computationally efficient chemical kinetic mechanisms. To improve the modeling capabilities of multicomponent fuels it is necessary to transition from the single-component descriptions of fuels and move toward more complex, accurate multicomponent ones, where different functionality groups are included. In this work a newly developed multicomponent reaction mechanism, capable of modeling the four different functionality groups *n*-, *iso*-, *cyclo*-alkanes and aromatics, is presented. The mechanism consists of seven fuel components with different molecular sizes and the mechanism offers the possibility to create surrogate fuels for real-world fossil and alternative fuels and fuel mixtures. The presented mechanism accurately models key combustion parameters at a wide range of conditions, considering the intrinsic characteristics of the four functional groups. The presented mechanism offers a unique combination of fuel flexibility, modeling performance and a low computational cost, and it opens up the possibilities to cost-efficient CFD simulations of multicomponent surrogate aviation fuels.

## 1 Introduction

Development of efficient and environmentally sustainable combustion devices require in-depth understanding of both chemical and physical aspects of the combustion process. A good understanding enables improved design of hardware, and fine-tuning of, for example, fuel injection. In addition, transition to renewable fuels with different properties compared to the traditional fossil fuels, and mixtures of renewable and fossil fuels, may require optimization of devices and processes.

To achieve a detailed understanding, it is convenient to combine experimental and numerical strategies, with a handful of properties being accessible from experimental studies and with more detailed understanding obtained using modeling.^1^ A significant challenge in combustion research is that the processes cover a vast range of temporal and spatial scales, including a large number of chemical species reacting at different time scales. There are large variations in local temperature, which induce a highly nonlinear behavior of chemical reactions. The properties accessible in experimental studies of turbulent combustion are the axial and radial velocities, temperature, concentrations of stable species and reactive radicals.^1^ However, species measurements are challenging and commonly a few selected species are measured, most often hydroxyl radical (OH) and formaldehyde (CH_2_O), and NO if the NOx pollutants are targets.

For the various submodels of a CFD simulation of reactive flow, the chemistry has a high priority in many cases,^2,3^ but its implementation is highly challenging due to the large number of species and chemical reactions. For many fuel molecules the knowledge is very good and predictions of pollutant formation and flame stability can be highly accurate. There is, however, still limited understanding of combustion and soot formation of larger hydrocarbons.^1^

Modeling of combustion of heavy real-world fuels such as diesel or aviation kerosene is particularly challenging from the chemical kinetics perspective. These fuels present two levels of complexity: the mixture composition and the size of the molecules. The fuel mixture consists of hundreds or even thousands of different hydrocarbons of different size and functionality: straight chained, branched, cyclic and unsaturated, in the size range from around C_4_ for the smaller gasoline compounds up to around C_20_ for the heavy diesel and kerosene components. The oxidation of such large hydrocarbons includes hundreds of important intermediate species and hundreds or even thousands of chemical reactions. Including both levels of complexity, a chemical kinetic mechanism would potentially include hundreds of thousands of chemical reactions.

The mixture complexity can be reduced in two ways, the greatest simplification is when the whole complexity is replaced with one single fuel molecule with average properties representing the fuel mixture, as applied in the HyChem suite of mechanisms^4,5^ and for several complex fuels by Zettervall et al.^6,7^ The second option is to use a surrogate mixture of about 2–10 compounds that represent different groups of components in the fuel mixture and the average properties of which agree with the average properties of the fuel. Surrogates methodologies have been outlined by, among others, Pitz and Mueller^8^ and Dooley et al.,^9,10^ and surrogate mixtures have been developed for gasoline,^11^ diesel^12^ and jet fuel.^13,14^

The challenge of the second level of complexity, the molecular size, is approached using mechanism simplification or reduction, meaning that the species and reactions with minor contribution to the oxidation process are removed or simplified by lumping them together. Reduced chemical mechanisms can broadly be divided into two groups; those where the sequence of main hydrocarbon intermediates are kept intact but minor reaction pathways are removed, and those where the pathway is simplified by lumping species and reactions to shorten the reaction sequence. To achieve compact mechanisms of heavy fuels, computationally cheap enough for CFD, the second approach is necessary. The successful approaches for heavy fuels build on decoupling of different parts of the kinetic mechanism, with various levels of detail in different subsets of the chemistry. This has allowed construction of compact kinetic mechanisms for 1-component fuels as applied by the HyChem group^5^ and Zettervall et al.,^6,15^ but also for multicomponent mechanisms of diesel and biodiesel by Chang et al.^16,17^ and for a range of FAME fuels by Zettervall et al.^18,19^

While the single fuel component approach has been successfully applied in many CFD simulation studies, it is not sufficient to achieve a complete understanding of the combustion process. The single component approach is useful when the fuel composition is well characterized, but for studies of the effect of changing fuel composition a surrogate fuel approach is necessary. An example of this can be studies of various amounts of drop-in fuel in a fossil kerosene. For this it is essential to be able to use the same mechanism but with different amounts of individual fuel components. Recent works presenting multicomponent has not had this broad scope, as can be exemplified by the 4-component mechanism by Saraee et al.^20^ who used a decoupling methodology to provide a compact chemical mechanism for jet A. Several recent works on multicomponent mechanisms for jet fuels include works on RP-3: Liu et al.^21^ present a 4-component mechanism with 153 species and 858 reactions; Wang et al.^22^ model mixtures of RP-3 and HEFA and claim to have produced a compact mechanism but without revealing the size; Xi et al.^23^ presents a 3-component RP-3 mechanism with only 43 species and 136 reactions.

In the present work we advance the use of highly compact mechanisms following our earlier works on single component^6,15^ and multicomponent^18^ heavy fuels. For the first time we here present a 7-component surrogate fuel mechanism including fuel components of varying functionalities, including *n*-alkane, *iso*-alkane, *cyclo*-alkane and aromatic. This mechanism is developed to be useful as surrogate for different fossil-based jet fuels, novel synthetic fuels, and the mixtures of these fuels. The mechanism is unique in that the surrogate compounds can be combined in different ways to enable use of the same mechanism to model several fuels and fuel mixtures. To our knowledge such a versatile but still highly compact mechanism has not been presented before.All single-component fuels treats chemistry for the averaged fuel molecule as if it is an *n*-alkane. The main challenge in the present mechanism development is that two of the new functional groups, the *cyclo*-alkane and aromatic, require new (highly compact) C_5_ and C_4_ submechanisms. This approach still conforms to the bottom-up modeling approach^19^ but results in a larger number of intermediate submechanisms, species and reactions compared to the single-component fuel mechanisms. The approach used in the modeling of the presented 7-component mechanism is described in detail in the section 2 below, followed by validation of single component performances, and finally multicomponent mixtures of real-world fuels.

## 2 Mechanism Development

### 2.1 Selection of Fuel Components

As outlined in the introduction, successful surrogate fuel mechanisms need to build on well-studied fuel components with similar chemical functionality as the real fuel components. A key property strongly related to molecular structure is the ignition delay time, which for some fuels have an exponential decrease in ignition delay times in correspondence to increases in temperatures, but for others show a so-called Negative Temperature Coefficient (NTC) trend at low to intermediate temperatures. Including *iso*-, *n-* and *cyclo*-alkanes, together with two aromatics, in the mechanism enables simulating a wide range of surrogate mixtures, with either long ignition delay times or strong NTC behaviors.

In the present work four common *iso*- and *n*-alkanes, with well-defined combustion characteristics are selected: *i*-C_8_H_18_ (*iso*-octane), *n*-C_10_H_22_ (*n*-decane), *i*-C_12_H_26_ (*iso*-dodecane) and *n*-C_12_H_26_ (*n*-dodecane). These are in a size range that make them suitable as surrogates components in mechanisms for diesel, but are also included in surrogates for aviation fuels.^24,25^

*cyclo*-Alkanes are represented by decalin, C_10_H_18_, also known as bicycle decane. Decalin is found in two forms, cis or trans, but the decalin species treated in this study does not differentiate between the two.

Two aromatic compounds are included, the monocyclic trimethylbenzene (C_9_H_12_ or TMBENZ) and the bicyclic methylnaphtalene (C_10_H_7_CH_3_, or MN). The significantly lower reactivity of aromatics compared to the alkanes results in lower CN of around eight to nine^26^ and a weaker NTC behavior. Here the trimethylbenzene molecule do not differentiate between 1,3,5- or 1,2,4-trimethylbenzene since they have common characteristics and almost identical ignition delay times.^27^ MN is used to define the lowest cetane number (CN) of 0.^26^

The seven species chosen in this mechanism means that surrogates representing a wide range of diesel and aviation fuels can be constructed, and the wide range of carbon sized species (from C_8_ to C_12_) enables surrogates of considerably different averaged molecular sizes to be constructed.

The challenge in constructing a compact and reliable mechanism for the selected fuel components lies in that they have quite different breakdown chemical mechanisms, both for the fuel breakdown steps and the intermediate chemistry. In particular, they are known for having widely different NTC behavior. The aromatic MN has a high C/H ratio and a close to linear trend in ignition delay time (i.e., no NTC), *i*-C_8_H_18_ and *i*-C_12_H_26_ both has an onset of NTC trend around 900 K (at 10 atm), while the *n*-alkanes *n-*C_10_H_22_/*n-*C_12_H_26_ has an onset of NTC trend above 1000 K.

### 2.2 Basic Development Methodology

The mechanism development starts with choosing a relevant set of reactions, and reaction rate parameters. The mechanism presented here uses the same set of C_2_ and H/C/O reactions as first introduced in previous mechanisms for propane,^3^ ethylene^28^ and JP-5/kerosene,^6,29^ and later modified for JP-10.^15^

The main combustion characteristics that the reduced mechanism is designed to predict are the laminar burning velocity, the flame temperature, the ignition delay time and the concentrations of major species (CO_2_, H_2_O, CO, H_2_), all for a wide range of equivalence ratios and initial gas temperatures and pressures. Guided by results from primarily experiments, and secondarily by simulation results from a detailed mechanism (C17790 mechanism^30^ with 492 species and 17,790 reactions), the proposed mechanism is tuned to fit these results by adjustments of rate parameters. The main reactions whose rates are adjusted are H + O_2_ → OH + O, C_2_H_4_ → C_2_H_3_ + H and C_2_H_3_ → C_2_H + H_2_, together with activation energies for reactions of the type C_*x*_H_*y*–1_ + O_2_ → C_*x*_H_*y*–1_O_2_, which is present for the *n*-, *iso*- and *cyclo*-alkanes. It should be noted that the adjustments to the pre-exponential factor or the activation energy are small and could be considered to be within uncertainty limits of these reactions.

The mechanism structure is divided in the blocks fuel breakdown, intermediate hydrocarbons (C_2_) and base mechanism (H/C/O).^19^ The basic strategy is that the large fuel molecule is quickly reduced into smaller hydrocarbons, C_6_, C_2_ and C_1_ for the *iso*- and *n*-alkanes, C_6_, C_4_, C_2_ and C_1_ for the *cyclo*-alkane, and C_5_, C_4_, C_2_ and C_1_ for the aromatics. These smaller hydrocarbons connects the fuel breakdown block to the underlying C_2_ and H/C/O blocks, which are common for all fuels. The intermediate hydrocarbons block consists of 5 C_2_ species and 22 irreversible reactions, and the base mechanism consists of 18 species and 43 irreversible reactions. The fuel breakdown blocks for each of the four alkanes include 7 unique species and 12 irreversible reactions. The block for decalin consists of 12 irreversible reactions and 7 species. For TMBENZ the block consist of 14 reactions, two of which are reversible, and 6 species. Finally the fuel breakdown block for MN include 6 species and 13 irreversible reactions.

All in all the mechanism has 71 species, 151 irreversible and two reversible reactions, and is from here on called Z153.

### 2.3 Fuel Breakdown

The modeling of the fuel breakdown of the four C_8_–C_12_ alkanes uses an approach first demonstrated by Chang et al.,^16^ and later modified by Zettervall et al.^19^ to work for even smaller reduced reaction mechanisms of large hydrocarbon molecules. The reactions are available in Table 1 and a summary description of the approach is presented in Section 2.3.1. Decalin follows a similar strategy, as outlined in Section 2.3.2.

**Table 1:** Reactions Used in the Fuel Breakdown Subsets for Each of the Seven fuel Molecules

*i***-C**_**8**_**H**_**18**_












***n*-C**_**10**_**H**_**22**_












***i*-C**_**12**_**H**_**26**_**/ *n*-C**_**12**_**H**_**26**_












**Decalin**












**Trimethylbenzene**














**MN**













**C**_**5**_**H**_**11**_**CO**


Due to the aromatic structure of TMBENZ and MN, additional C_5_ and C_4_ submechanisms are needed. These submechanisms, presented in Sections 2.3.3 and 2.3.4, are inspired by reactions and reaction rate parameters employed in the C17790 mechanism.

#### 2.3.1 *iso*- and *n*-Alkanes

The initial attack on the fuel molecule consists of H-abstraction reactions via either reactive radicals (H, OH) or more stable species (O_2_, HO_2_), to produce fuel radicals
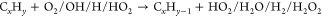
R1

For the three alkanes the same reaction rate parameters are used for the four H-abstracting reactions. The fuel radicals are in the next step decomposed at high temperatures via either thermal decomposition
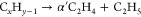
R2Or H-abstraction
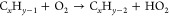
R3Here the newly formed radical C_*x*_H_*y*–2_ reacts with O_2_ to from smaller C_2_ and C_1_ species. At lower temperatures the fuel radical instead undergoes O_2_-addition
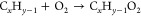
R4

In reaction R2 α′ = 3, 4, and 5 for *i*-C_8_H_18_, *n*-C_10_H_22_ and *i*-C_12_H_26_/*n*-C_12_H_26_, respectively. Previous studies have shown the that C_2_H_4_ is the dominating intermediate hydrocarbon at higher temperatures,^31,32^ hence the majority of the product species in reaction R2 are C_2_H_4_. For the low temperature reaction path, the following sequence takes place
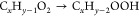
R5
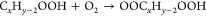
R6
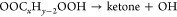
R7

The ketone is then oxidized into formaldehyde, C_5_H_11_CO and C_2_ and C_1_ species, and further oxidize C_5_H_11_CO to form C_2_H_5_, C_2_H_4_, CH, CO and HO_2_ (see Table 1).

#### 2.3.2 Decalin

Decalin follows similar fuel breakdown but with additional routes for the formation of C_4_H_4_ from the fuel radical via a thermal decomposition reaction. A higher C/H ratio results in the production of higher-order C species such as C_4_H_4_. With the formation of C_4_H_4_ comes the requirement for reactions associated with this additional species. The reaction for C_4_H_4_ and the subsequent species formed C_4_H_3_ and C_4_H_2_ is described below in the section detailing the modeling of MN.

#### 2.3.3 Trimethylbenzene

A recent publication by Dong et al.^33^ on the low-temperature reaction pathways for 1,2,4-trimethybenzene shows that this low CN aromatic exhibits NTC behavior, albeit significantly less pronounced compared to the *iso*- and *n*-alkanes.

The modeling of trimethylbenzene presented in this study is a highly reduced version of the one presented by Dong et al.^33^ The initial H-abstraction of the fuel comes from any of the six species O_2_, O, OH, H, HO_2_ or CH_3_, via
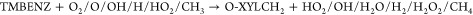
R8

The fuel radical follows either a low-temperature route with O_2_-addition
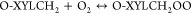
R9

Or a high-temperature route represented by either of the two following reactions,
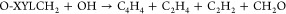
R10
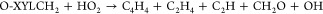
R11

As in the case of decalin the trimethylbenzene also includes C_4_H_4_. Following on the low-temperature route initiated by reaction R9 leads to the reactions
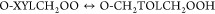
R12
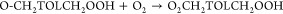
R13
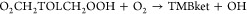
R14

Finally, the TMBket is thermally decomposed via
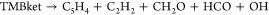
R15

Reactions R14 and R15 produces significant amounts of OH, accelerating the reactivity of the system at lower temperatures. As seen the overall structure of the fuel decomposition of trimethylbenzene is similar to the ones for the alkanes, but there are some minor differences. First, reactions R9 and R12 are both reversible, the only two reversible reactions in Z153. Keeping reactions R9 and R12 as reversible was key in order to keep the high modeling capacity of the low-temperature chemistry presented by Dong et al.^33^ On top of that, reactions R9 and R13 are both modeled as pressure dependent reactions. Reaction R15 produces C_5_H_4_, a species that represents a high carbon containing radical which can be present in the oxidation of high C/H ratios molecuels such as trimethylbenzene. As with the C_4_H_4_ molecule the reactions for C_5_H_4_ will be presented in the section of the fuel breakdown of MN below.

#### 2.3.4 Methylnaphtalene

Methylnaphthalene oxidation does not include the oxygen addition step typical for low-temperature chemistry, and therefore does not exhibit a NTC behavior for its ignition delay time. Similarly as in high-temperature mechanisms for JP-5/kerosene^29,34^ the emphasis is on thermal decomposition of the fuel as a first step, R16, and with a central role of the H atom in the oxidation of intermediates C_10_H_7_, C_5_H_4_, C_4_H_4_ (see Table 1) and C_4_H_3_. The modeling of the reduced reaction pathways uses similar reactions and reaction rate parameters as in the C17790 mechanism which includes a detailed set of species and reactions for the modeling of MN.
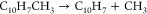
R16

C_10_H_7_ is then oxidized through reactions with either O_2_ or H
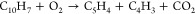
R17
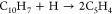
R18

The C_5_H_4_ species again reacts with either O_2_ or H
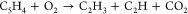
R19
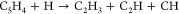
R20
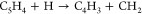
R21

Forming either C_4_H_3_ that needs to oxidize in the C_4_H_3_ submechanism, or directly C_2_ and C_1_ species. This means that C_10_H_7_ produces C_4_H_3_ by either reaction R17, or via the route (R17) → (R21). C_4_H_3_ enters the C_4_H_3_ submechanism to form either C_4_H_4_ or C_4_H_2_, through
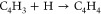
R22
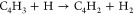
R23
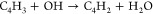
R24

C_4_H_4_ reacts back into C_4_H_3_ whereas C_4_H_2_ oxidizes to C_2_ and C_1_ species
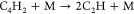
R25
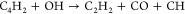
R26

Reactions R25 and R26 end in C_2_ and C_1_ species, finishing the MN fuel breakdown block and coupling MN to the underlying C_2_ and C_1_ blocks.

## 3 Results and Discussion

Mechanism validation will be presented for single components, binary mixtures, and realistic examples modeling surrogate fuel mixtures representing highly complex fuels. For the latter both Z153 and C17790 has appropriate surrogate formulations. For all results, Z153 will be compared to experimental data and results from the detailed reaction mechanism, and all will be evaluated for a wide range of equivalence ratios and pressure and temperature conditions. In order to keep the result section from becoming too extensive, simulation results for several pressure and temperature conditions are placed in the Supporting Information, as is simulation results for maximum flame temperatures.

All simulations has been performed using the Cantera software, version 2.4.0.^35^ The laminar burning velocity simulations, simulated on a one-dimensional grid, uses grid-refinement strategies, with an initial grid length of 1.5 cm. As an example, the grid refinement results in a final number of grid points of around 780 points for Z153, and 730 points for C17790, when simulating *n*-C_12_H_26_ at initial conditions of *p* = 1 atm and *T* = 400 K.

For the ignition delay time simulations a constant volume assumption is used, an assumption that best correlates with the set-ups of the experimental data at lower temperatures. At higher temperatures, both constant volume and constant pressure assumptions generate similar ignition delay time results. The simulation definition of the ignition delay time is when the temperature has increased 300 K above the initial temperature. This definition roughly corresponds to when the OH* is at its maximum,^28^ a common approach to define ignition in experiments, but the definition is also a good estimate that matches other experimental ignition definitions.

Thermodynamic and transport data has been collected from the database of Goos et al.^36^ and previously presented mechanisms, such as the mechanism by Dong et al.,^37^ Chang et al.^16^ and Yao et al.^38^

### 3.1 Single Components

#### 3.1.1 Laminar Burning Velocity

**Figure 1:**
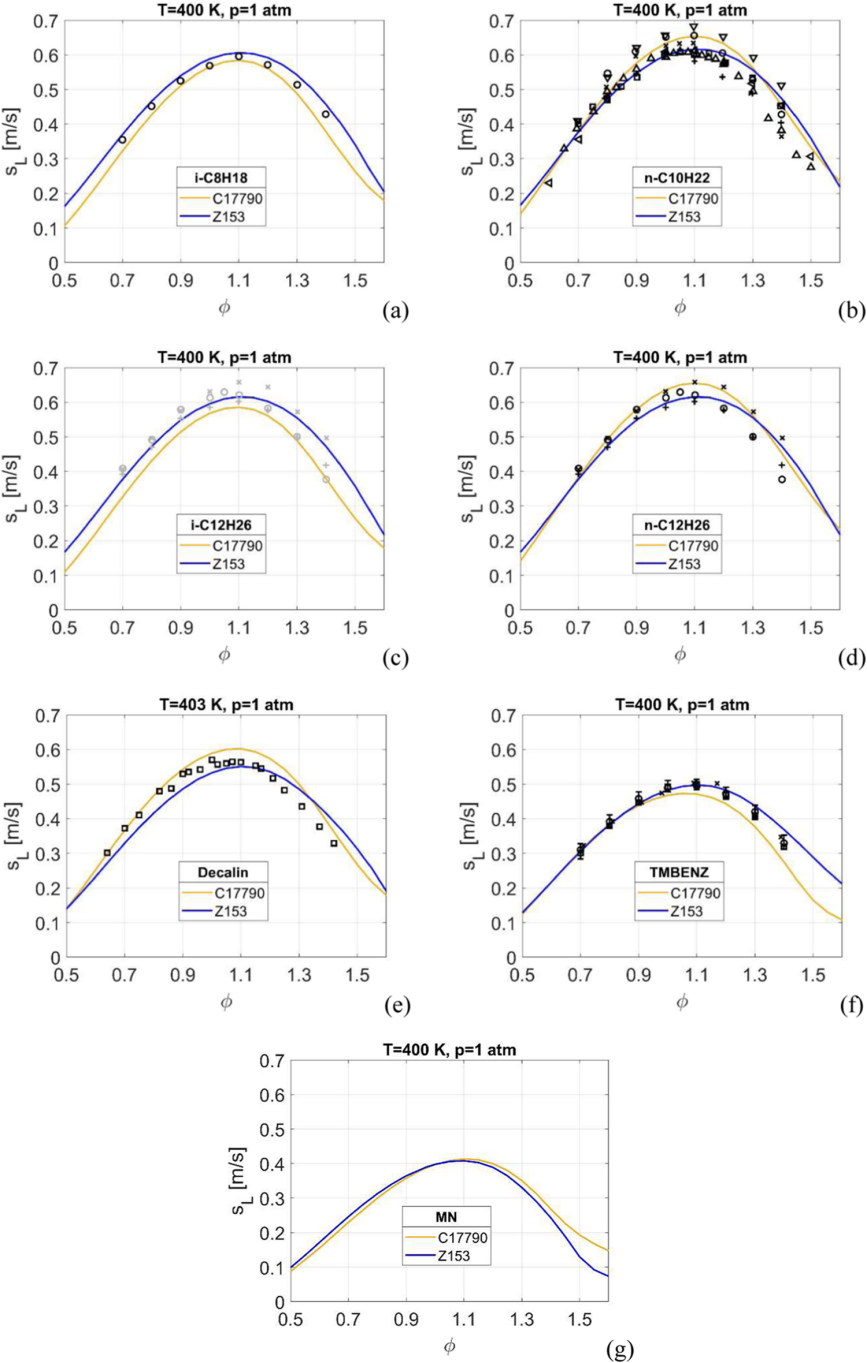
Laminar burning velocity at *p* = 1 atm and *T* = 400 K for (a) *i*-C_8_H_18_; (b) *n*-C_10_H_22_; (c) *i*-C_12_H_26_; (d) *n*-C_12_H_26_; (e) Decalin; (f) TMBENZ; and (g) MN. Experimental data: in (a): o -;^40^ in (b): o -,^41^ x -,^39^ □ -,^42^ v -,^40^ < -,^43^ + -,^44^ ∧ -;^45^ in (c), data for *n*-C_12_H_26_: o -,^39^ x -,^40^ + -;^41^ in (d): o -,^39^ x -,^40^ + -;^41^ in (e): □ -;^46^ in (f): o -,^47^ □ -,^47^ x -.^48^

Laminar burning velocities for all seven fuels, at ambient pressure and *T* = 400 or 403 K, are shown in Figure 1. All four *iso*- and *n*-alkanes show similar burning velocities, which is expected,^39^ with maximum burning velocities of around 60 cm/s. The burning velocities gradually decreases with the *cyclo*-alkane (decalin) and the aromatics, with MN having significantly lower (∼50%) velocities compared to the *iso*- and *n*-alkanes. The laminar burning velocity is not only dependent on carbon number but also on molecular structure, and this is clearly shown in the cases of trimethylbenzene and MN and their significantly lower burning velocities.

The availability of experimental data differs significantly between the fuels, with no data available for *i*-C_12_H_26_, data for *n*-C_12_H_26_ is used instead in Figure. 1c, and for MN.

Overall, the burning velocities simulated by Z153 is in good agreement with all experimental data present, and to the reference mechanism, for all seven fuels over a wide range of equivalence ratios. The same is also true at elevated initial gas temperatures and pressures, see Supporting Information.

#### 3.1.2 Ignition Delay Time

**Figure 2:**
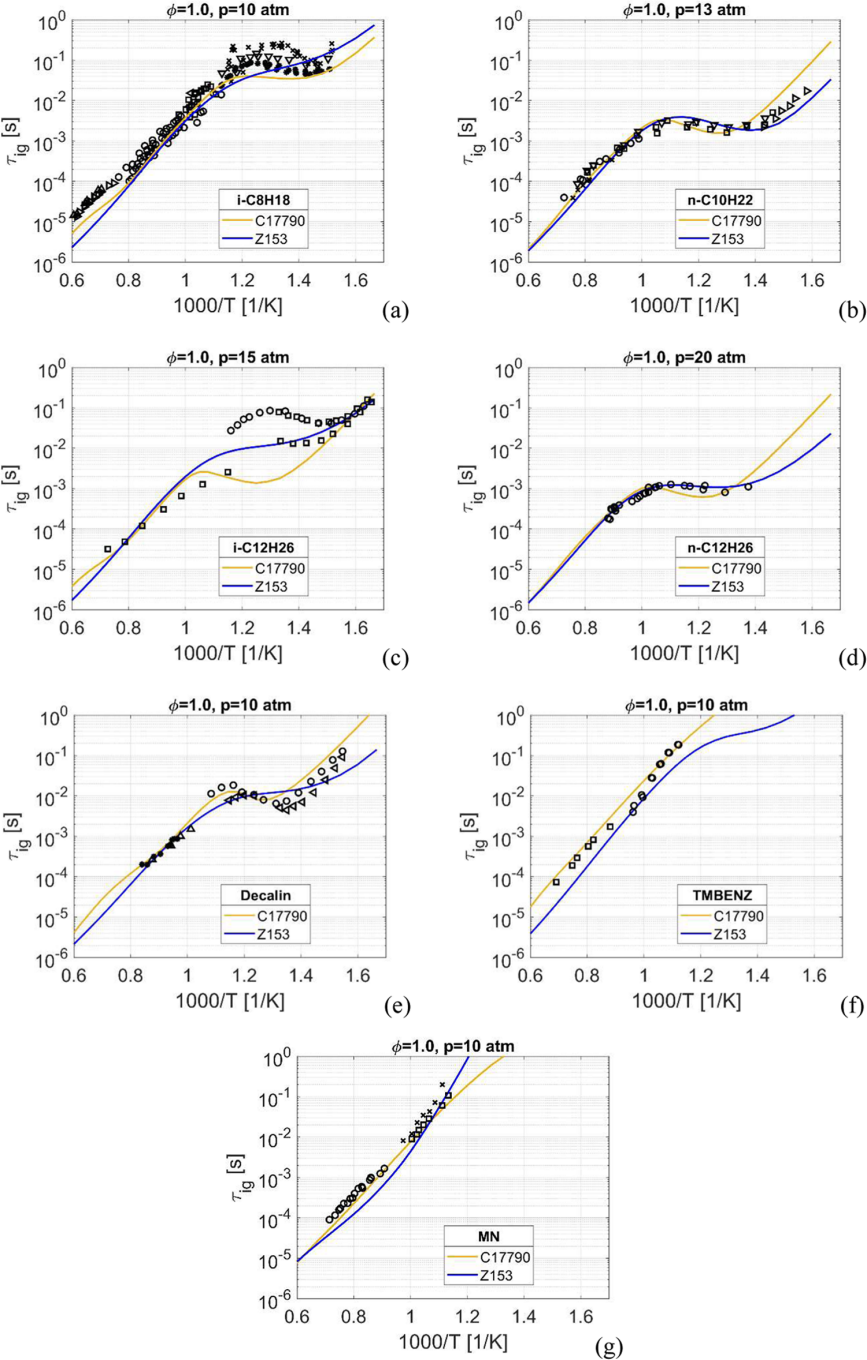
Ignition delay time, at stoichiometric conditions. *i*-C_8_H_18_ at *p* = 10 atm in (a), *n*-C_10_H_22_ at *p* = 13 atm in (b), *i*-C_12_H_26_ at *p* = 15 atm in (c), *n*-C_12_H_26_ at *p* = 20 atm in (d), decalin at *p* = 10 atm in (e), trimethylbenzene at *p* = 10 atm in (f) and C_10_H_7_CH_3_ at *p* = 10 atm in (g). Experimental data: in (a): x -,^49^ o -,^50^ > -,^51^ □ -,^52^ ∧ -,^53^ v -,^54^ * -,^55^ < -;^56^ in (b): x -,^57^ □ -,^58^ > -,^59^ o -,^60^ v -;^58^ in (c): □ -,^61^ o -;^62^ in (d): o -;^63^ in (e): o -,^64^ ∧ -,^65^ < -,^66^ * -;^67^ in (f): o -,^33^ □ -;^48^ in (g): x -,^68^ □ -,^68^ o -.^69^

**Figure 3:**
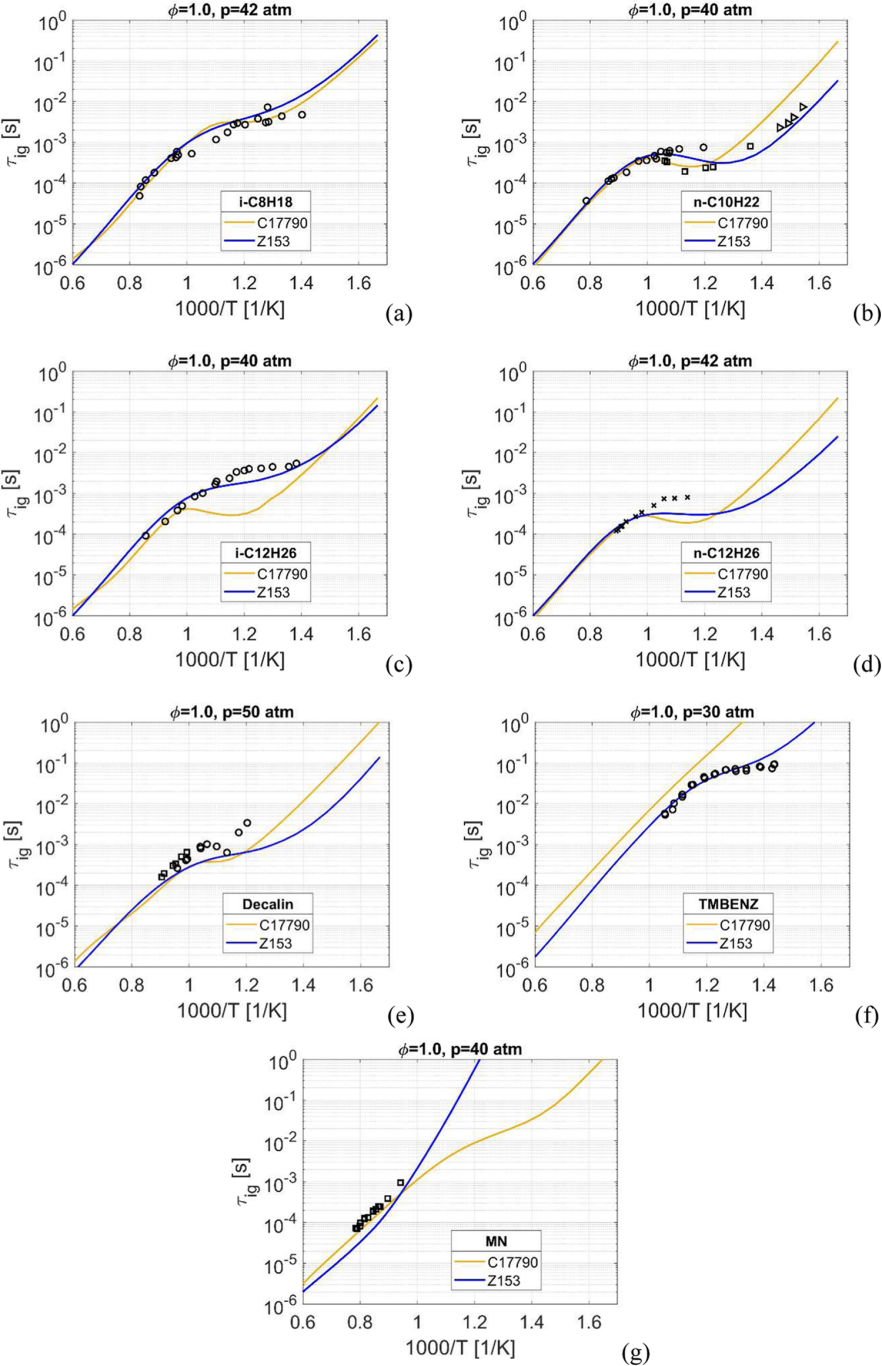
Ignition delay time, at stoichiometric conditions. *i*-C_8_H_18_ at *p* = 42 atm in (a), *n*-C_10_H_22_ at *p* = 40 atm in (b), *i*-C_12_H_26_ at *p* = 30 atm in (c), *n*-C_12_H_26_ at *p* = 42 atm in (d), decalin at *p* = 50 atm in (e), trimethylbenzene at *p* = 30 atm in (f) and C_10_H_7_CH_3_ at *p* = 40 atm in (g). Experimental data: in (a): o -;^70^ in (b): o -,^60^ □ -,^58^ > -;^59^ in (c): o -;^61^ in (d): x -;^63^ in (e): o -,^65^ □ -;^67^ in (f): o -;^33^ in (g): □ -.^69^

The ignition delay time is modeled for stoichiometric, fuel lean and fuel rich conditions, at a wide range of temperatures and for pressures ranging from ambient to up to 50 atm. The results presented here are for a medium-pressure case, between 10 to 20 atm, shown in Figure 2, and a high-pressure case, at 30 atm or higher, shown in Figure 3, all at stoichiometric conditions. Results for other equivalence ratios and pressures are available in the Supporting Information.

At medium pressures, 10 to 20 atm, Figure 2a-d, the agreement to the experimental data is good for the four *iso*- and *n*-alkanes, for both Z153 and C17790. The NTC of Z153 is slightly more pronounced than that of C17790 for the two *n*-alkanes, resulting in a better agreement with the experimental data for lowest temperatures. For both *iso*-alkanes in Z153, i-C_8_H_18_ and i-C_12_H_26_, no NTC is present, however the ignition delay times are leveling off at the lower temperatures, and the simulation results fall within the range of the experimental data. The reason for the absence of a NTC behavior for both *iso*-alkanes is the slower reaction rate of reaction R4. Note the significant difference in ignition delay times, especially below 1000 K, between the *iso*-alkanes and the *n*-alkanes. At higher temperatures the differences are significantly smaller with both the *iso*-alkane and the two *n*-alkanes converging toward similar values, which the mechanisms capture quite well.

For the *cyclo*-alkane decalin in Figure 2e the NTC behavior is relatively strong, resembling the NTC behavior of the *n*-alkanes but with longer ignition times. Here both mechanisms matches the experimental data well, with C17790 having significantly stronger NTC behavior compared to Z153.

Moving on to the aromatics the CN falls significantly and the NTC behavior is almost extinguished. At *p* = 10 atm for trimethylbenzene, Figure 2f, low-temperature data is not present, so it is not possible to determine what is an accurate level of NTC behavior. However, as will be seen later, higher pressures will demonstrate the NTC behavior that trimethylbenzene actually exhibits.

The ignition delay times of MN, Figure 2g, show a complete lack of NTC characteristic. At 1000 K the ignition delay time of MN is roughly one order greater than for the *iso*-, *n*- and *cyclo*-alkanes and although no experimental data exists below 800 K the trend in the existing data shows no indication of a NTC behavior. This lack of NTC is also supported by the CN of zero. For MN Z153 and C17790 show diverging behaviors at around 1200 K, where Z153 has an upward trend in the ignition curve whereas C17790 shows a flattening curve shape. These trends are seen even more clearly for some of the initial conditions presented in the Supporting Information. Below around 1200 K the ignition delay times are in excess of 1 s making them irrelevant for many combustion applications and these long ignition times highlights the need for inclusion of faster igniting *n*-alkanes.

When moving from medium-pressure conditions in Figure 2 to high-pressure conditions, *p* ≥ 30 atm in Figure 3, the overall trend is faster ignition. For the *iso*- and *n*-alkanes in Figures 3a–d the two mechanisms show similar results, except for *i*-C_12_H_26_ at around 800 K where C17790 again has a too fast ignition with a pronounced NTC.

For decalin in Figure 2e both mechanisms show good agreement to around 1000 K, after which Z153 has faster ignition compared to the experimental data. For trimethylbenzene, Figure 3f, Z153 matches the low-temperature data very well whereas C17790 completely lacks any NTC behavior.

For MN, Figure 3g, the diverging trend between Z153 and C17790 below 1000 K is accentuated compared to the medium-pressure case in Figure 2g, and the curve trend of C17790 almost resembles a weak NTC behavior. At *p* = 10 atm the diverging behavior started at around 800 K but at these higher pressures the divergence has increased and starts at 1000 K. Unfortunately no experimental data exists below 1000 K but based on the trends at 10 (Figure 2g) and 15 atm (Supporting Information) Z153 can be considered to have an overall closer resemblance to experimental data compared to C17790.

### 3.2 Multi Component

To further investigate the cocombustion of the aromatic MN and alkanes, in the following two subsections, ignition delay time for mixtures is reported. This is followed by use of the mechanism for cases where some of the seven components are included to mimic real fuel mixtures.

#### 3.2.1 *n*-C_12_H_26_/C_10_H_7_CH_3_ Mixtures

**Figure 4:**
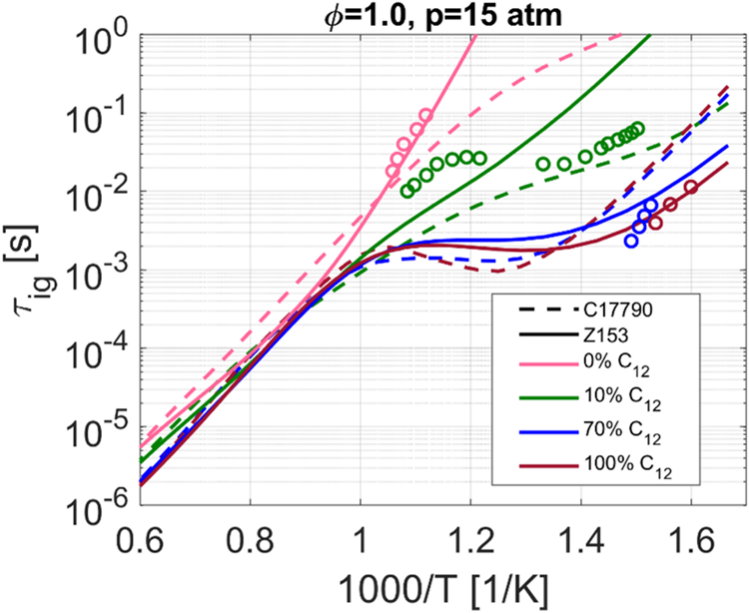
Ignition delay time, *p* = 15 atm and φ = 1.0, using various *n*-C_12_H_26_/MN mixtures. Solid lines for Z153 and dashed for C17790.

A recent paper from Kukkadapu and Sung^71^ reported ignition delay times for MN/*n*-C_12_H_26_ mixtures, at *p* = 15 atm, ranging from 0 to 100% *n*-C_12_H_26_. All-in-all, seven mixtures were experimentally investigated and simulation results using four of those mixtures are presented in Figure 4. All four mixtures in this figure is color coded with the results from Z153 shown in solid lines and C17790 in dashed lines. The four mixtures chosen are two with low amounts of *n*-C_12_H_26_ addition (0 and 10%), and the two with high amounts (70 and 100%).

With 10% molar percentage of *n*-C_12_H_26_, green color, the measured ignition delay times clearly show a NTC behavior, not captured by either of the kinetic mechanisms. Z153 predicts ignition delay times somewhat in the middle of the experimental data set whereas C17790 predicts a too fast ignition. Based on the satisfactory results for *n*-C_12_H_26_ previously presented in Figures 2d and 3d, and for the 100% *n*-C_12_H_26_ mixture in Figure 4, the slow ignition of the methylnaphthalene clearly has an inhibitory effect on the NTC behavior of the MN/*n*-C_12_H_26_ mixture in the two mechanisms. Accurately capturing the NTC effect at 10% *n*-C_12_H_26_ is a major challenge, and not even the highly chemically complex C17790 mechanisms manages this. At 70% *n*-C_12_H_26_, blue color, both mechanisms display a significant NTC behavior with Z153 being in slightly better agreement with the experimental data. Note that the NTC behavior is similar when 70 and 100% of the mixture consists of *n*-C_12_H_26_ indicating that even if 30% of the mixture consists of a CN = 0 fuel the ignition delay time is relatively unaffected.

Ignition delay time is dominated by the rapid radical production from the fuel that ignite the fastest and having 70% *n*-alkanes is more than enough to dominate the ignition behavior.

#### 3.2.2 *n*-C_10_H_22_/C_10_H_7_CH_3_ Mixtures

Experimental data for ignition delay times for 30/70 and 70/30 compositions of *n*-C_10_H_22_/MN, at 10 ant 40 atm, was presented by Frassoldati et al.^72^ where the mixtures were used as diesel surrogates.

**Figure 5:**
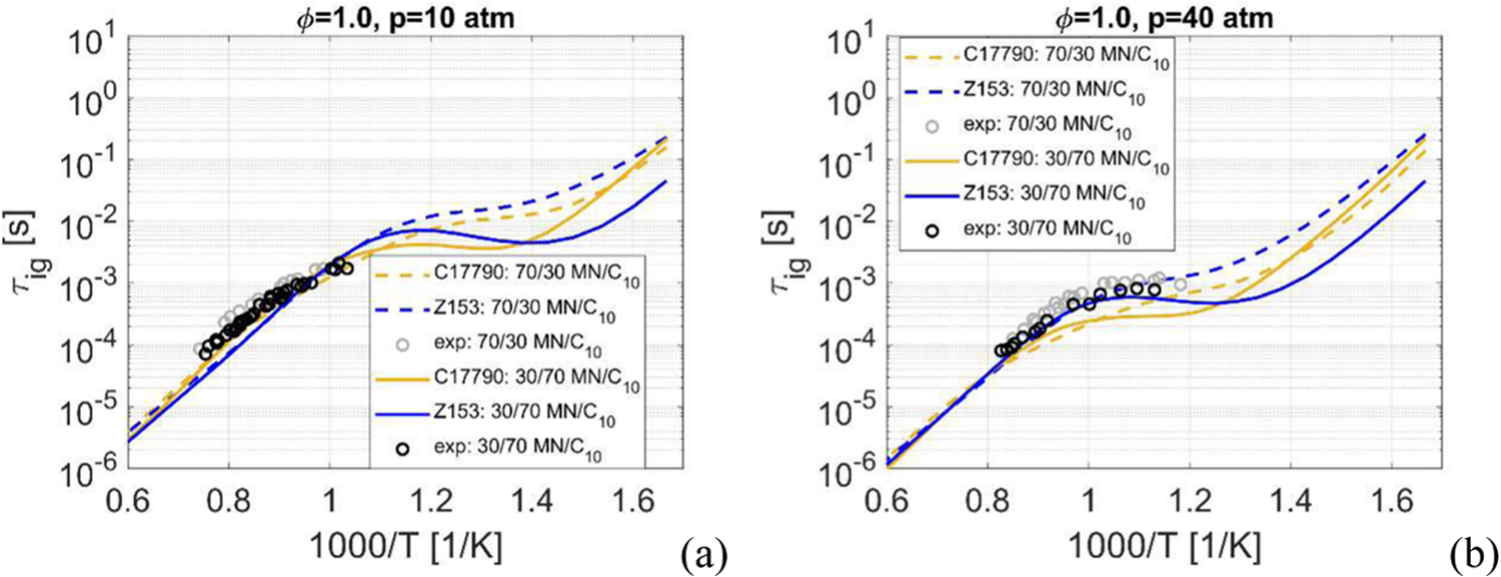
Ignition delay time, *p* = 10 atm and φ = 1.0 in (a) and *p* = 40 atm and φ = 1.0 in (b), using *n*-C_10_H_22_/MN: 30/70 and 70/30 mixtures.

Simulation results for Z153 and C17790 for 30/70 and 70/30 *n*-C_10_H_22_/MN compositions are shown in Figure 5, at 10 atm in Figure 5a and 40 atm in Figure 5b. Overall, the results for both mechanisms, at both mixtures compositions and for both pressures agrees well with the experimental data, with the exception of C17790 at the higher pressure and below 1000 K where it has a too fast ignition prediction. Higher MN content results in longer ignition delay times, mainly below 1000 K, and higher pressures results in shorter overall ignition delay times, as expected.

Note that Z153 has a slightly stronger NTC behavior compared to C17790 for mixtures with 70% *n*-C_10_H_22_ and this feature can be seen for the 100% *n*-C_10_H_22_ case above, Figures 2b and 3b. At 70% MN the results are reversed with C17790 having lower ignition delay times at lower temperatures. This is expected since the 100% MN predictions by the two mechanisms show that Z153 has a weaker flattening of the ignition curve compared to C17790, and at these high MN enrichments these inherent characteristics of the MN modeling in the mechanisms will be more pronounced. Lack of experimental data at low temperatures makes it impossible to know which mechanism is better.

### 3.3 Fuel Surrogates

To broaden the investigation of the modeling capabilities of Z153 this section will expand the multi component mixture concept to not only mix two species into one fuel blend but rather creating multi component surrogates of real fuels, both fossil and synthetic fuels. The two fuels chosen are the common Jet A, and the synthetic fuel C5, both undergoing detailed investigations in the Nation Jet Fuels Combustion Program.^73^ These two fuels have very different molecular compositions, a fact that is represented in the surrogates created here. Since the fuel breakdown blocks of all seven fuel species are independent of each other choosing the surrogate mixture is a separate process that does not demand any further chemical kinetic modeling or adjustments. The independence of the seven fuel breakdown blocks means that if only for example four species are used to represent the surrogate fuel the other three unused fuels can be removed, effectively minimizing the mechanism size and computational cost. The only time all 153 reactions are included is when all seven species are chosen in a surrogate mixture.

By creating fuel surrogates a more accurate description of a jet fuel or synthetic fuel can be created compared to when using the simpler approach of an averaged molecule. This in turn means that when a multicomponent surrogate is used to model a jet or synthetic fuel in a CFD a more detailed representation of the fuel can be had, and the intricate fuel process nuances can be modeled. One way where this can manifest itself is in the vaporization of liquid droplets where different fuel molecules can vaporize at different temperatures, times and locations, creating regions dominated by certain fuel molecules. Another possibility is a sequential ignition process where fuel molecules with highly different ignition characteristics ignites at different times and locations around a spray cloud. Individual fuel species transport properties can be modeled as well, determined by the inherent transport properties of the different fuel classes. None of this can be achieved by the simpler one-component averaged molecular strategy commonly used today, and Z153 represents a new, more accurate approach to the handling of jet fuels and synthetic fuels in CFD simulations.

#### 3.3.1 Jet A

Jet A is a conventional distillate jet fuel consisting of a wide variety of species. The investigation by Edwards^74^ shows that a Jet A composition consists of 20% by mass *n*-paraffins, 29.4% by mass *iso*-paraffins, 31.9% by mass *cyclo*-paraffins and 18.7% by mass aromatics. Each group is in the present work represented by one component: *n*-C_12_H_26_, *i*-C_12_H_26_, decalin and trimethylbenzene. This gives an average molecular component of C_10.8_H_20.4_, close to the measured C_11.4_H_21.7_, with molecular weights of 150.9 and 158.6 g/mol, respectively.

**Figure 6:**
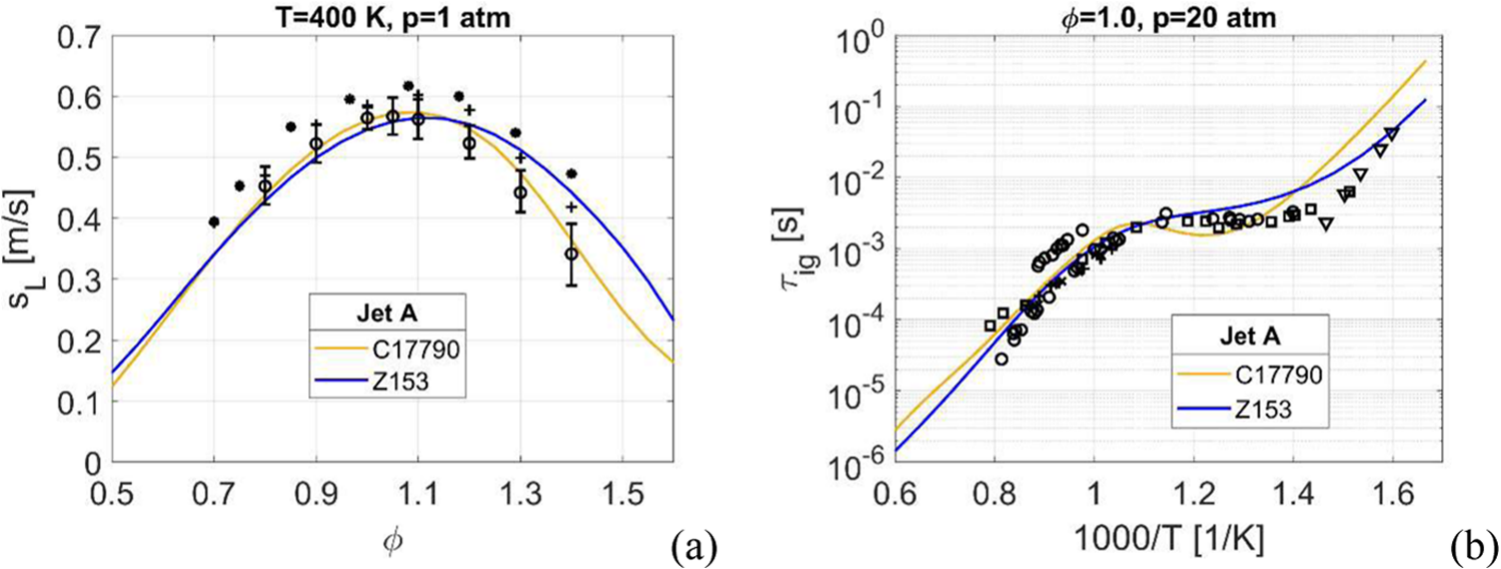
Laminar flame speed, (a), and ignition delay time, (b), for Jet A. The ignition delay times are at stoichiometric conditions and *p* = 20 atm. Experimental data: in (a): o -,^5^ * -,^75^ + -;^41^ in (b): o -,^76^ x -,^77^ v -,^78^ □ -,^79^ + -.^80^

The four-component Jet A surrogate is simulated using both Z153 and C17790, with the laminar flame speeds shown in Figure 6a, and the ignition delay time in Figure 6b. Initial gas temperatures, pressures and equivalence ratios are chosen based on the availability of experimental data. For the laminar flame speeds in 6a both mechanisms are in good agreement with the experimental data. Overall, both mechanisms matches the experimental data well.

#### 3.3.2 C5 Synthetic Jet Fuel

**Figure 7:**
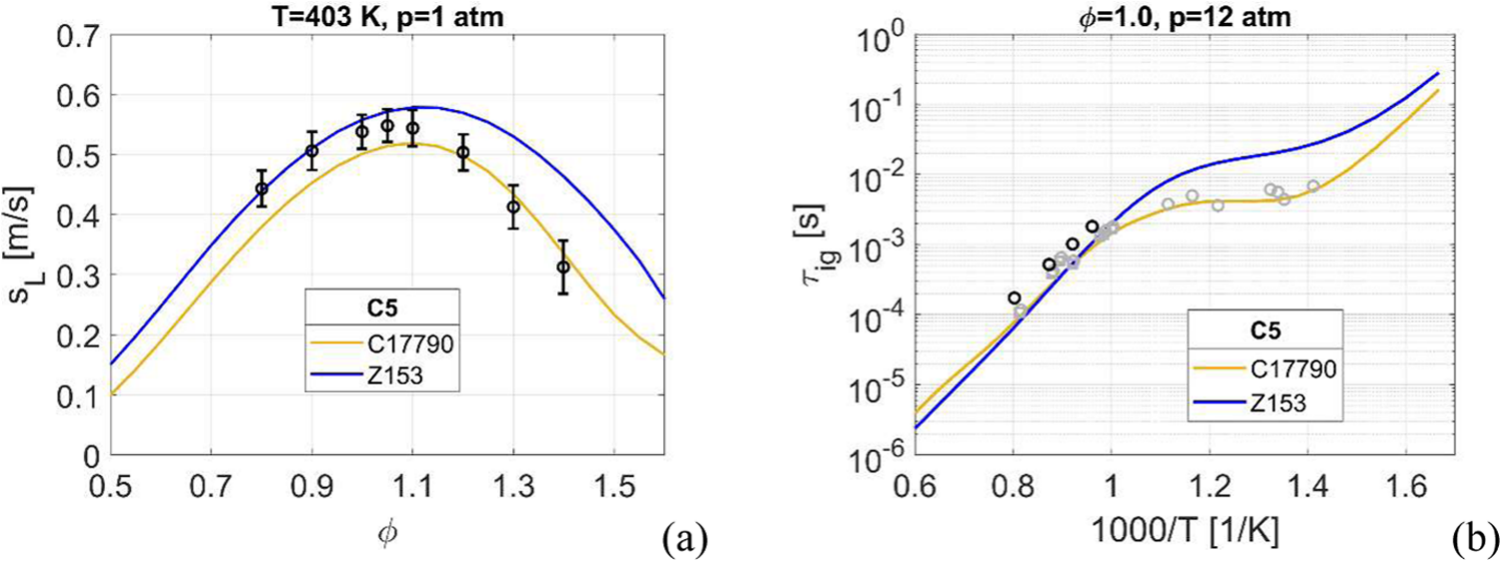
Laminar burning velocity, (a) and ignition delay time at *p* = 12 atm and φ = 1.0, (b). Experimental data: in (a): o - *; in (b): black circles - **, and gray circles for JP-5.^32^ Source: * A. Movagar, F. N. Egolfolooulos, personal communication (2017). ** D.F. Davidson, Y. Zhu, J. Shao, R.K. Hanson, personal communication (2016).

C5 is a model synthetic jet fuel with an average molecular formula C_9.7_H_18.7_, a molecular weight of 126.2 g/mol and a H/C ratio of 1.93. In contrast to many other jet fuels it has no *cyclo*-alkanes but is dominated by *iso*-alkanes (51.6% by mass), has a large aromatics content (30.7% by mass) with the remainder being *n*-alkanes (17.7% by mass).^81^ When creating a surrogate for C5 using the Z153 and C17790 mechanisms the composition of 25.8% by mass of i-C_8_H_18_, 25.8% by mass of i-C_12_H_26_, 17.7% by mass of *n*-C_10_H_22_ and 30.7% by mass of MN was used, resulting in a surrogate with an average molecule of C_10.3_H_18.4_, a molecular weight of 138.3 g/mol and a H/C ratio of 1.79. These properties closely mimic the real fuel and the surrogate composition was simulated at conditions where experimental data sets for C5 are available, see Figure 7.

For the laminar flame speed, Figure 7a, only one experimental data set exists with Z153 matching the data at fuel lean and stoichiometric conditions, and C17790 at fuel rich conditions.

When discussing the ignition characteristics of C5 it is important to recognize that both the composition of the fuel and its CN of 39.6^81^ indicate that there should indeed exist a NTC trend. This CN is close to that of the JP-5 fuel, CN = 39.1.^81^ Unfortunately, there are not yet any experimental data at low temperatures for C5, but modeling using the C17790 and Z153 mechanisms together with experimental data for JP-5 both indicates an NTC behavior. Both Z153 and C17790 using C5 surrogates show a clear NTC behavior, similar to the data for JP-5, included as gray symbols in Figure 7b. Without experimental data of the fuel in question it is impossible to be certain what level of NTC is correct for C5. At temperatures above 1000 K both mechanisms are in good agreement, matching the C5 experimental data (black symbols) well.

## 4 Conclusions

Z153 matches the experimental data for all seven fuel species, for all simulated cases.. It also matches the C17790 reference mechanism except for some cases of *i*-C_12_H_26_ and MN where the reference mechanism does not match the experimental data well. Z153 manages to match experimental data and simulation results by C17790 for bicomponent and multicomponent surrogate fuels, extending to both fossil-based fuels and synthetic fuels, exemplified here using Jet A and C5. A high modeling performance despite the compact format of Z153 shows that the modeling structure presented by Chang et al.^16^ and later modified by Zettervall et al.^6,19^ is highly effective, accurate and is applicable to a wide range of species.

The mechanism structure of Z153 is modular^19^ meaning that no fuel breakdown subsection is dependent on any other. This means that one only need to include the fuel breakdown subsections of the fuels of interest, keeping the number of species and reactions to a minimum, assuring a minimal computational cost.

The multifaceted nature of Z153 means that this single mechanism can be used to represent a wide variety of jet fuels and synthetic fuels, something that is normally only possible when using (computationally expensive) detailed multicomponent mechanisms.

## Supporting Information Available

Additional mechanism validation figures, including a short outline and references (PDF)
Chemical kinetic mechanism available in Chemkin and Cantera and YAML format (ZIP)
