# Supplementary material for: A Compact Chemical Kinetic Mechanism for Heavy Fuel Surrogates with n-, iso- and cyclo-Alkanes, and Aromatic Compounds
Source: ACS Omega. 2025 Apr 11;10(15):15471–83. doi: 10.1021/acsomega.5c00158 (PMC12019505; doi:10.1021/acsomega.5c00158)
Supplement: Supplementary file 1 — ao5c00158_si_001.pdf [file ao5c00158_si_001.pdf]

Supplementary Material for the article:

A compact chemical kinetic mechanism for heavy fuel surrogates with n-, iso- and cyclo-alkanes, and aromatic compounds

Niklas Zettervall[1], *Elna J. K. Nilsson*[2, *]

[1] Weapons, Protection and Security, Swedish Defence Research Agency FOI, 164 90, Stockholm, Sweden.

[2] Division of Combustion Physics, Lund University, Box 118, 22100 Lund, Sweden

Supplementary Material

Results for the ignition delay times and laminar burning velocities, at various initial gas temperature and pressures, and equivalence ratios. The choice of initial conditions simulated has been dictated by the availability of experimental data. The results below are divided into individual fuel species subsections.

For the ignition delay times the pressure ranges from p=1 atm (for trimethylbenzene) up to as high as p=50 atm (for n-$C_{10}H_{22}$), with the temperature range spanning from T=600 K up to 1667 K. Results show ignition delay times for both fuel lean, stoichiometric and fuel rich conditions.

For the laminar flame speeds two elevated pressures, p=3 and 10 atm, at T=400 K, are used for all seven fuel molecules. Simulations at elevated initial gas temperatures are dictated by the availability of experimental data and ranges from T=443 K (decalin), T=450 K (for n-$C_{12}H_{26}$ / i-$C_{12}H_{26}$, decalin) up to T=470 K (for i-C_8H_{18}, n-$C_{10}H_{22}$, n-$C_{12}H_{26}$ and methylnaphthalene).

In general, both Z153 and the C17790 mechanisms are in good to acceptable agreement with experimental data for all of the conditions simulated.

Results for i-C_8H_{18}

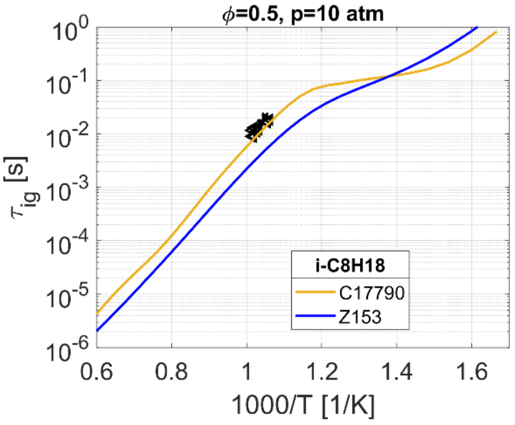

(a)

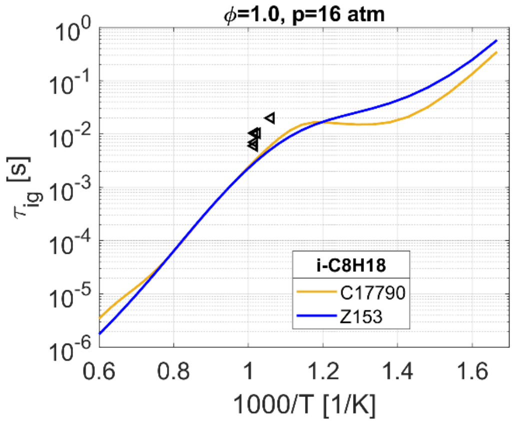

(b)

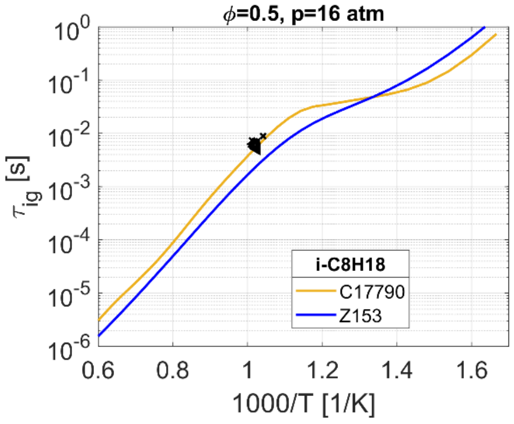

(c)

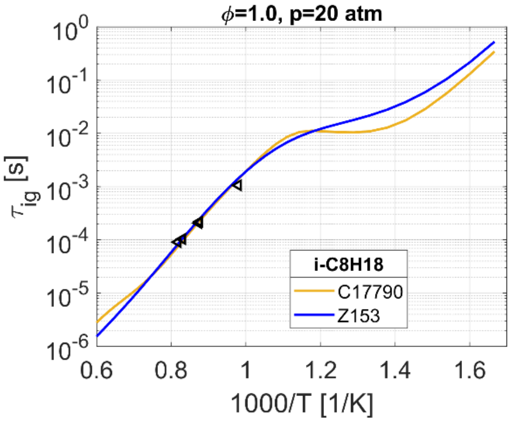

(d)

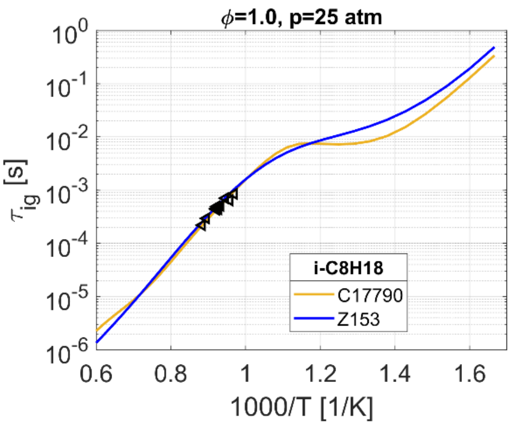

(e)

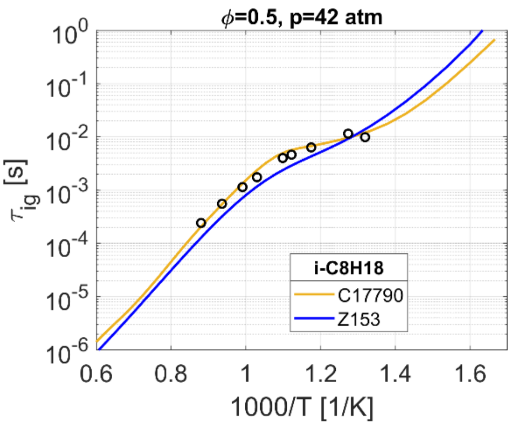

(f)

Figure S1. Ignition delay time of i-C_8H_{18} at p=10 atm and φ=0.5 in (a), p=16 atm and φ=1.0 in (b), p=16 atm and φ=0.5 in (c), p=20 atm and φ=1.0 in (d), p=25 atm and φ=1.0 in (e) and p=42 atm and φ=0.5 in (f). Experimental data: in (a): **<** - (1), **x** - (1); in (b): **<** - (1); in (c): **<** - (1), **x** - (1); in (d): **<** - (2); in (e): **<** - (2); in (f): **o** - (3).

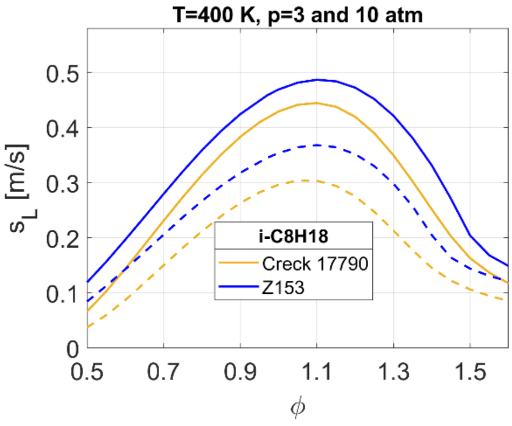

(a)

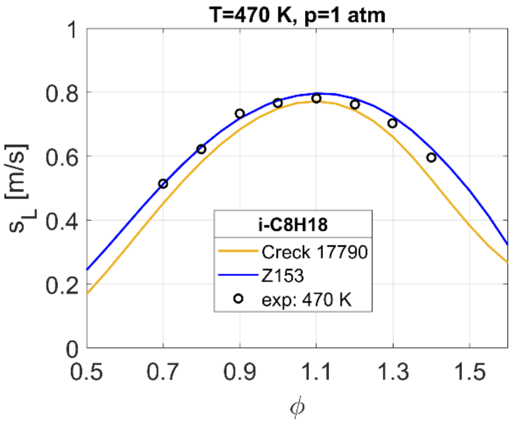

(b)

Figure S2. Laminar flame speed for T=400 K and p=3 and 10 atm in (a), and T=470 K and p=1 atm in (b). Experimental data: in (b): **o** - (4).

Results for n-$C_{10}H_{22}$

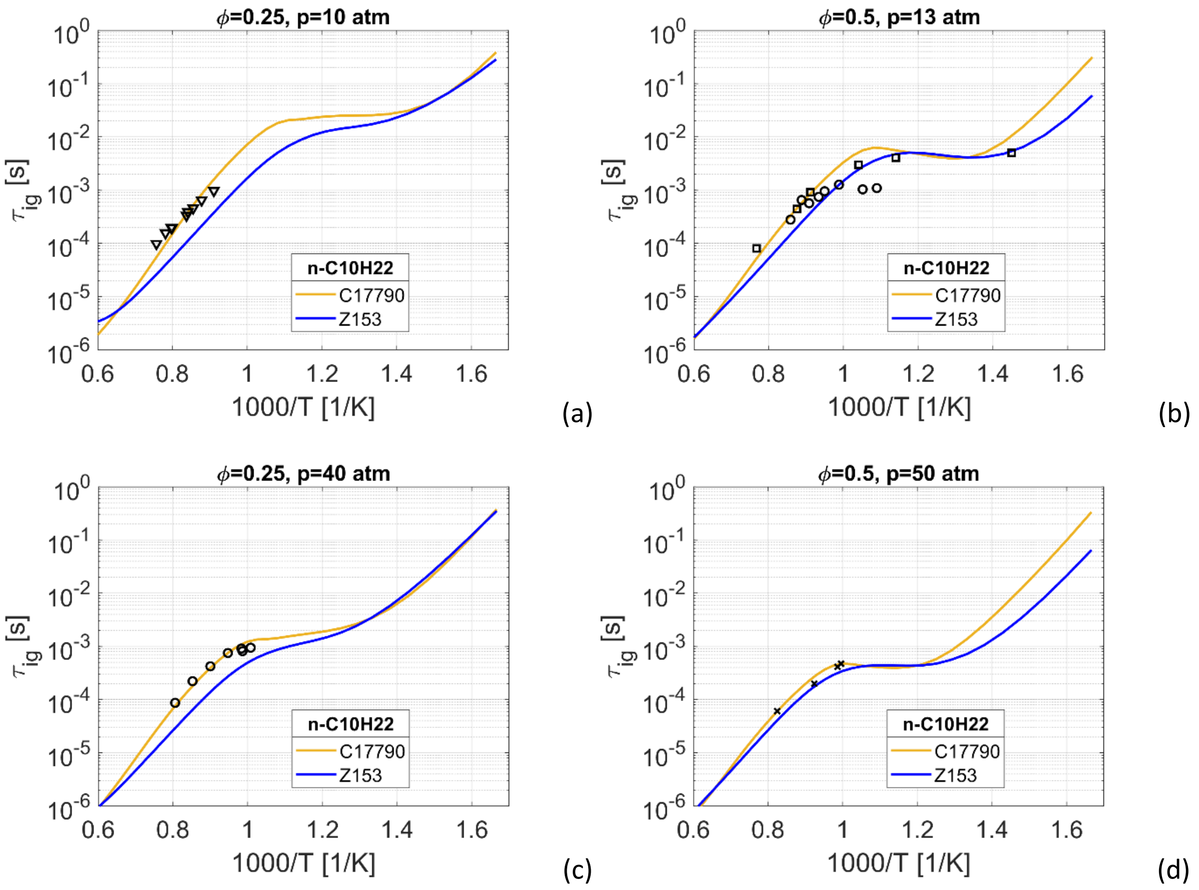

Figure S3. Ignition delay time of n-$C_{10}H_{22}$ at p=10 atm and φ=0.25 in (a), p=13 atm and φ=0.5 in (b), p=40 atm and φ=0.25 in (c) and p=50 atm and φ=0.5 in (d). Experimental data: in (a): **v** - (5); in (b): **o** - (5), □ - (6); in (c): **o** - (5); in (d): **x** - (5).

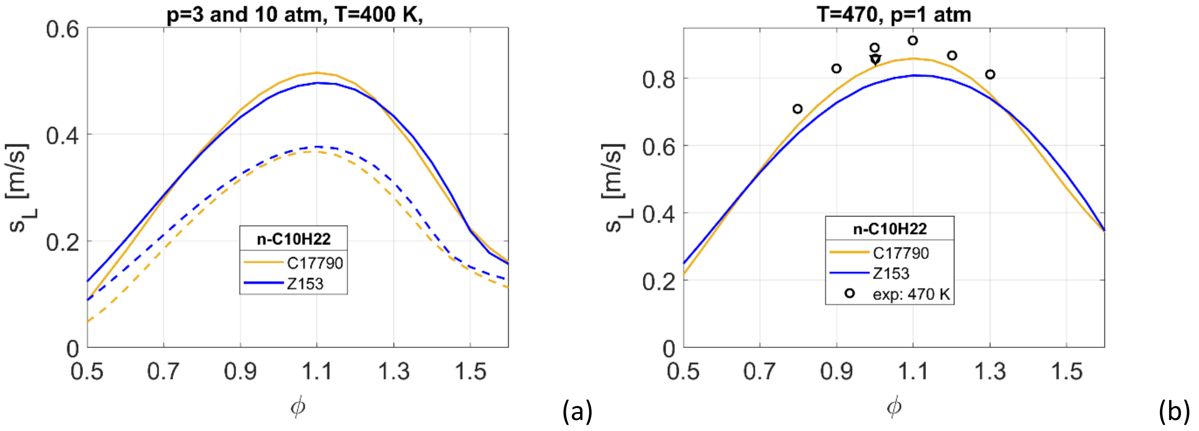

Figure S4. Laminar flame speed for T=400 K and p=3 and 10 atm in (a), and T=470 K and p=1 atm in (b). Experimental data: in (b): **o** - (7), **v** - (8).

Results for i-$C_{12}H_{26}$

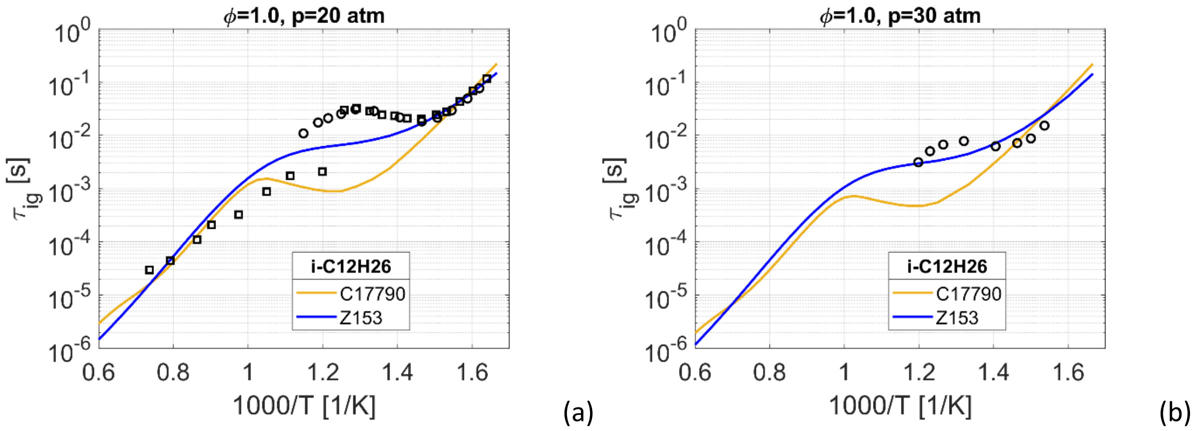

Figure S5. Ignition delay time of i-$C_{12}H_{26}$ at p=20 atm and φ=1.0. Experimental data: in (a): **o** - (9), □ - (9), in (b): **o** - (10).

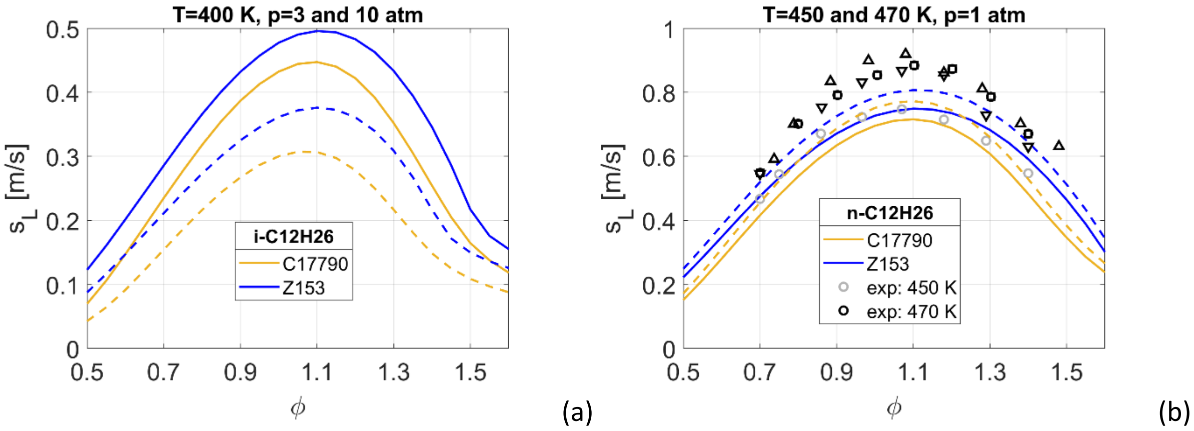

Figure S6. Figure X. Laminar flame speed for T=400 K and p=3 and 10 atm in (a), and T=470 K and p=1 atm in (b). Experimental data (for n-$C_{12}H_{26}$): in (b): **^** - (11), **o** - (7), **o** - (grey symbols) - (11).

Results for n-$C_{12}H_{26}$

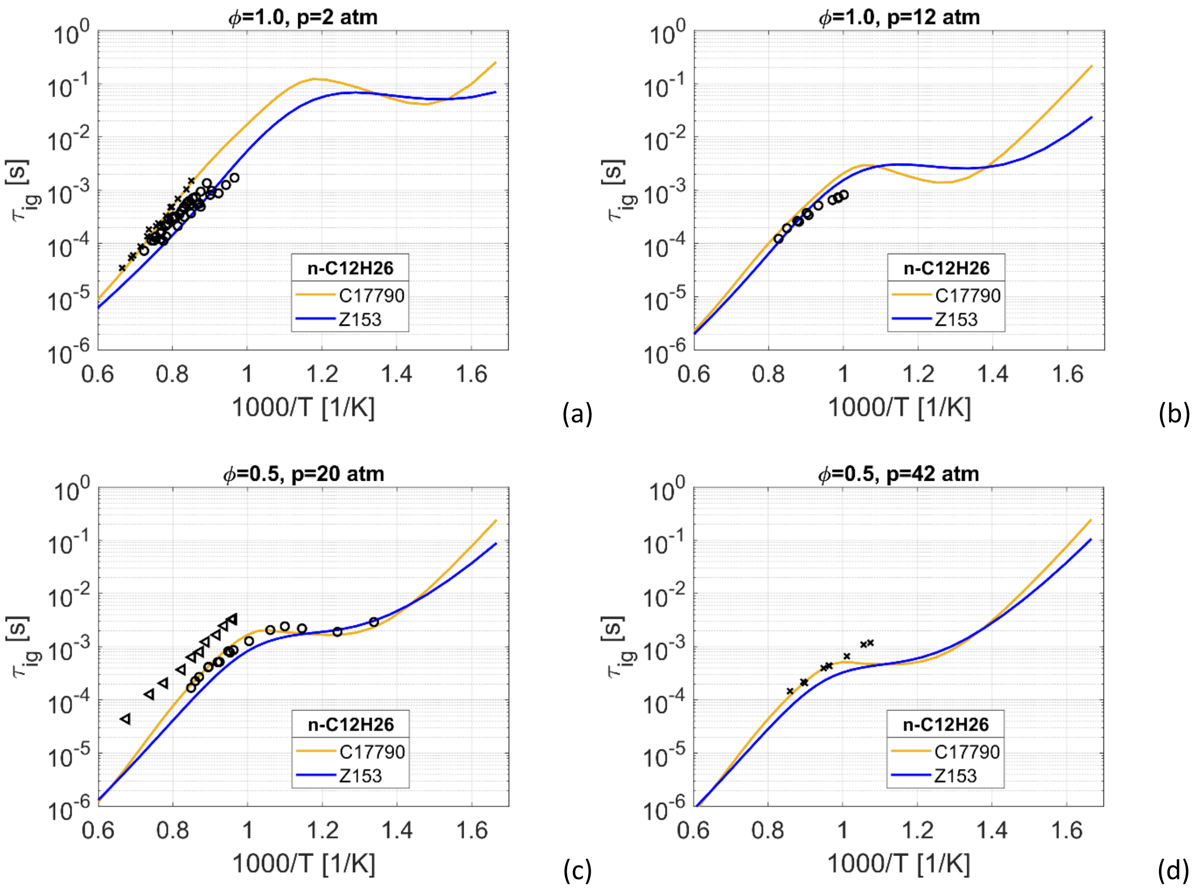

Figure S7. Ignition delay time of n-$C_{10}H_{22}$ at p=2 atm and φ=1.0 in (a), p=12 atm and φ=1.0 in (b), p=20 atm and φ=0.5 in (c) and p=42 atm and φ=0.5 in (d). Experimental data: in (a): **o** - (12), **x** - (13); in (b): **o** - (5); in (c): **<** - (14), **o** - (15); in (d): **x** - (5)**.**

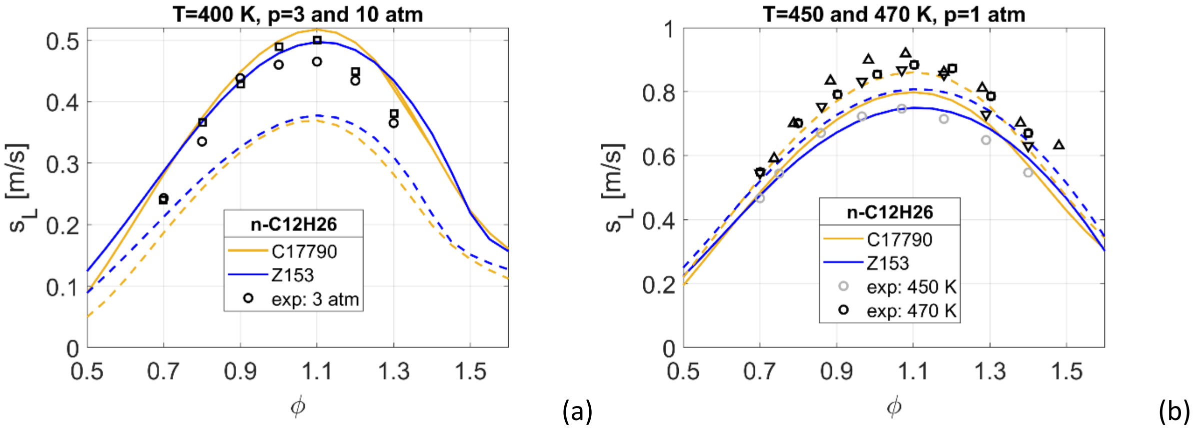

Figure S8. Laminar flame speed for T=400 K and p=3 and 10 atm in (a), and T=470 K and p=1 atm in (b). Experimental data: in (a): **o** - (8), □ - (16); in (b) **^** - (17), **v** - (11), **o** - (7), **o** - (grey symbols) - (11):

Results for decalin

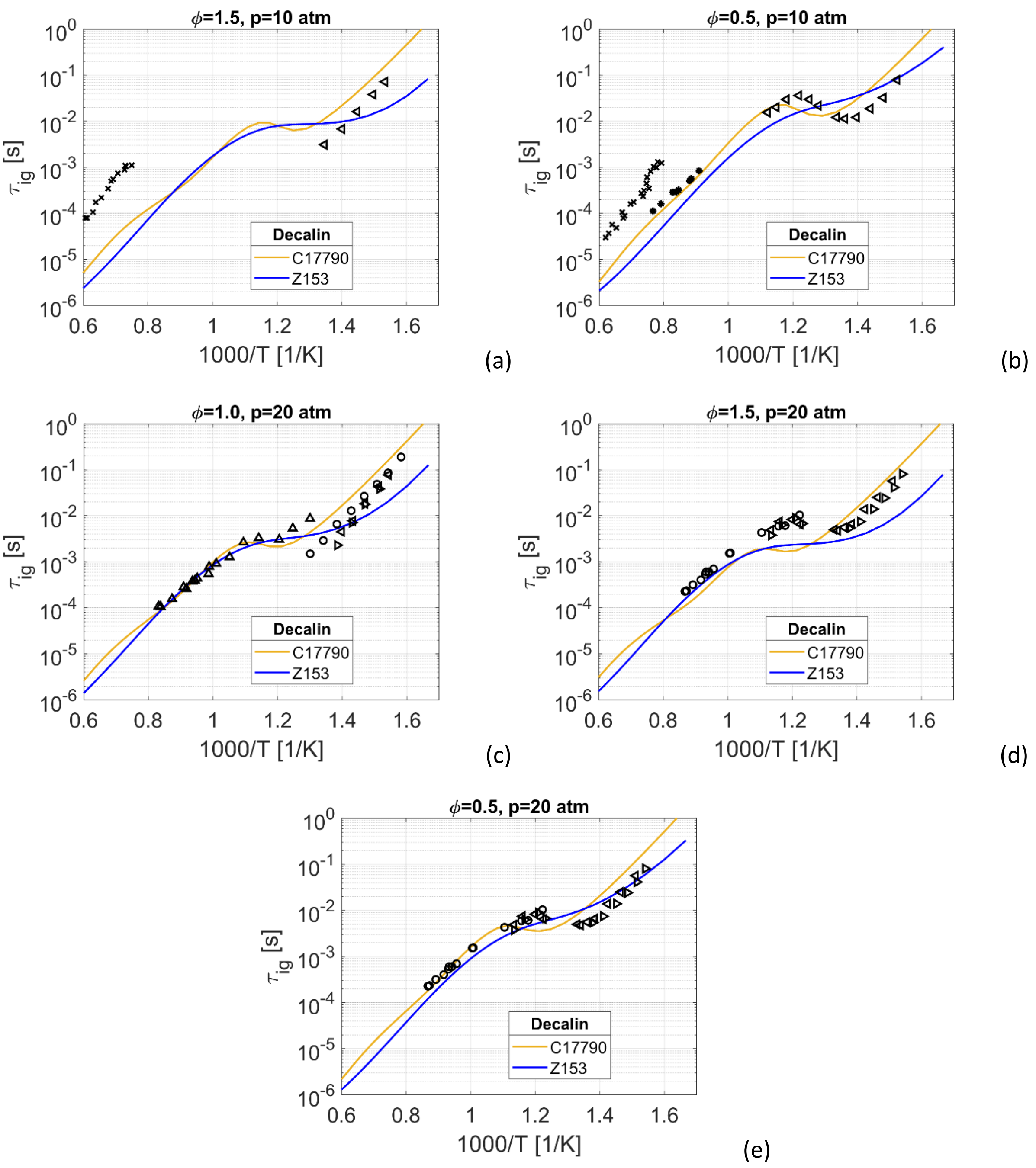

Figure S9. Ignition delay time of decalin at p=10 atm and $\varphi=0.5$ in (a), p=20 atm and $\varphi=1.0$ in (b), p=20 atm and $\varphi=1.5$ in (c) and p=20 atm and $\varphi=0.5$ in (d). Experimental data: in (a): **<** - (18), **x** - (19); in (b): **<** - (18), **x** - (19), * - (20); in (c): **^** - (21), **o** - (22), **>** - (18), **<** - (18); in (d): **o** - (21), **>** - (18), **<** - (18); in (e): **o** - (21), **>** - (18), **<** - (18).

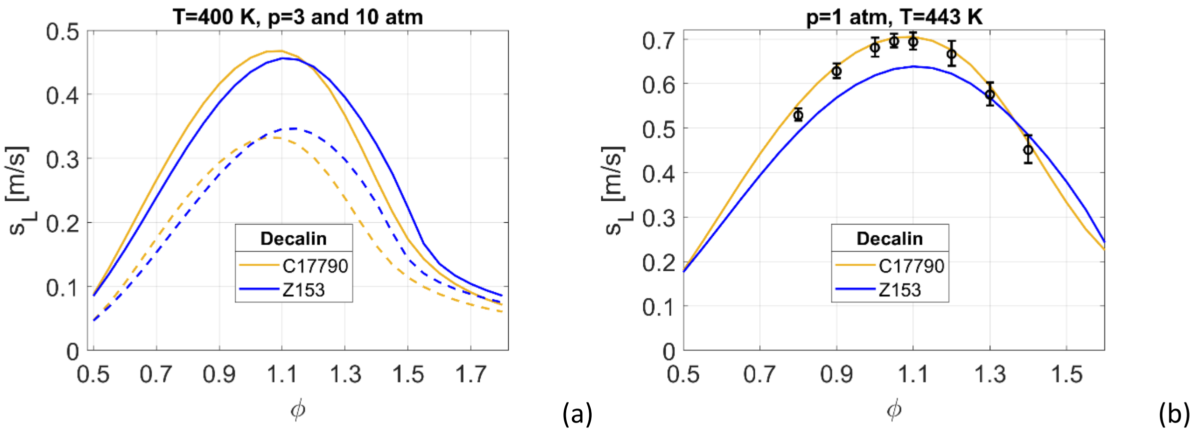

Figure S10. Laminar flame speed for T=400 K and p=3 and 10 atm in (a), and T=443 K and p=1 atm in (b). Experimental data: in (b): **o** - (23).

Results for trimethylbenzene

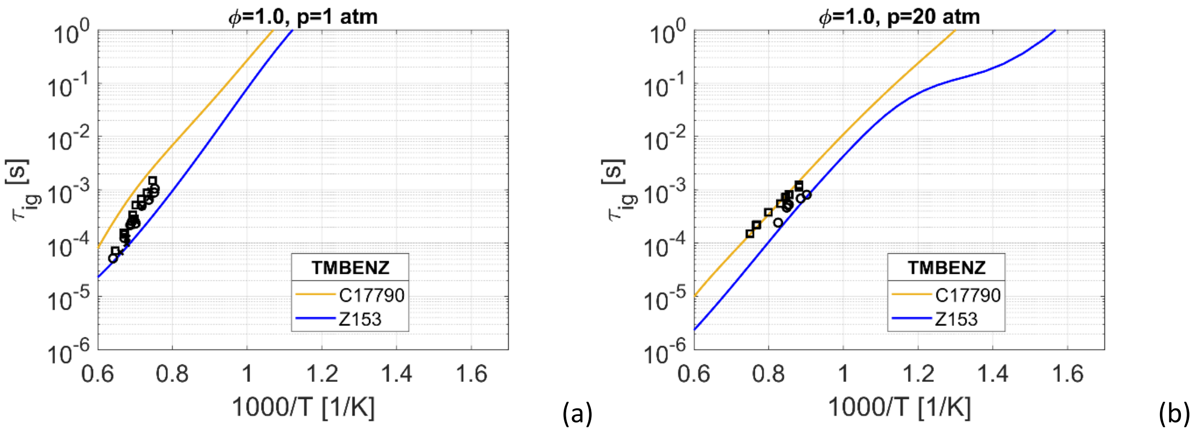

Figure S11. Ignition delay time of trimethylbenzene at p=1 atm and φ=1.0 in (a), p=20 atm and φ=1.0 in (b). Experimental data: in (a): **o** - (24), **x** - (24), □ - (24); in (b): **o** - (24), □ - (25).

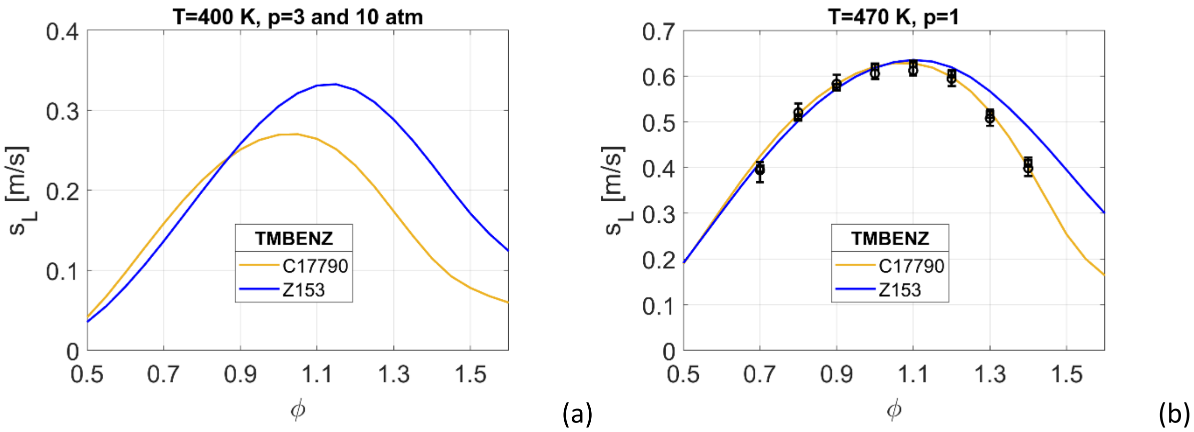

Figure S12. Figure 8. Laminar flame speed for T=400 K and p=3 and 10 atm in (a), and T=470 K and p=1 atm in (b). Experimental data: in (b): **o** - (26).

Results for $C_{10}H_7CH_3$

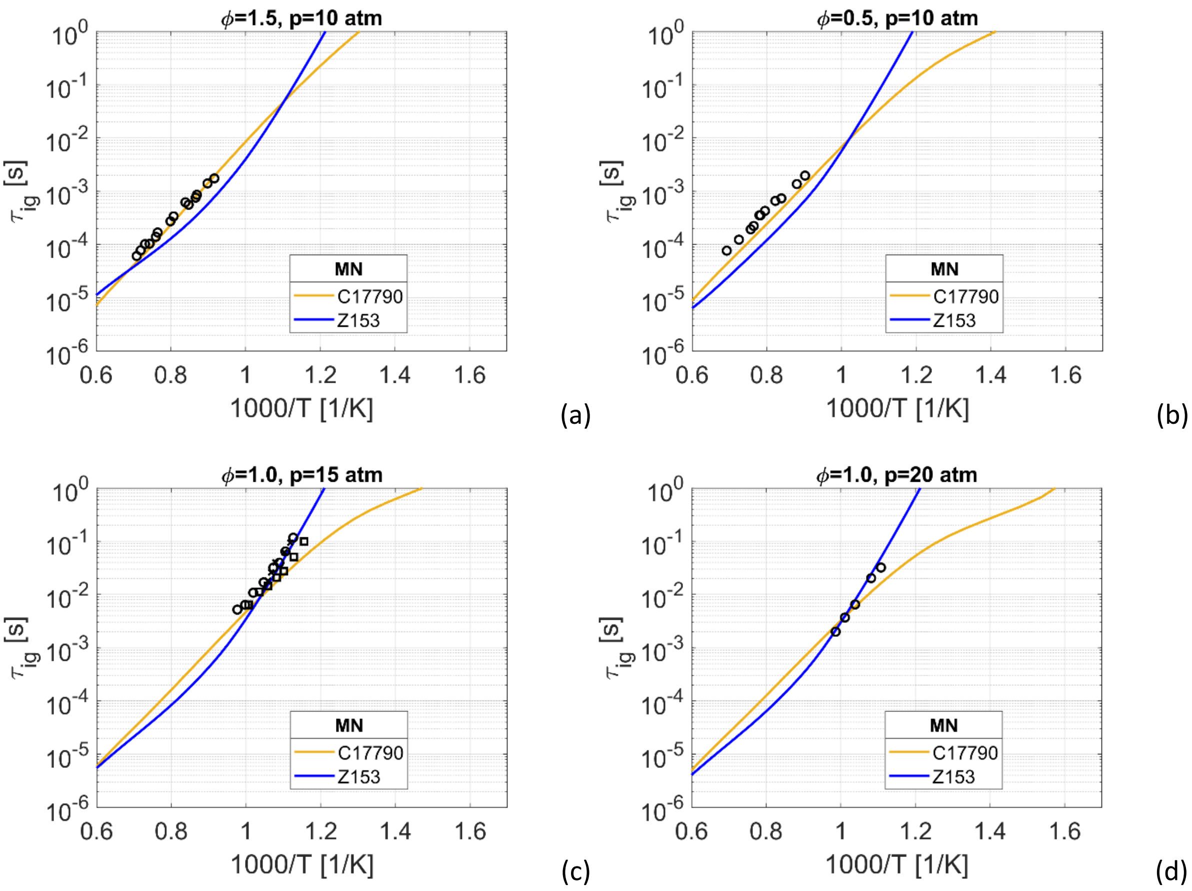

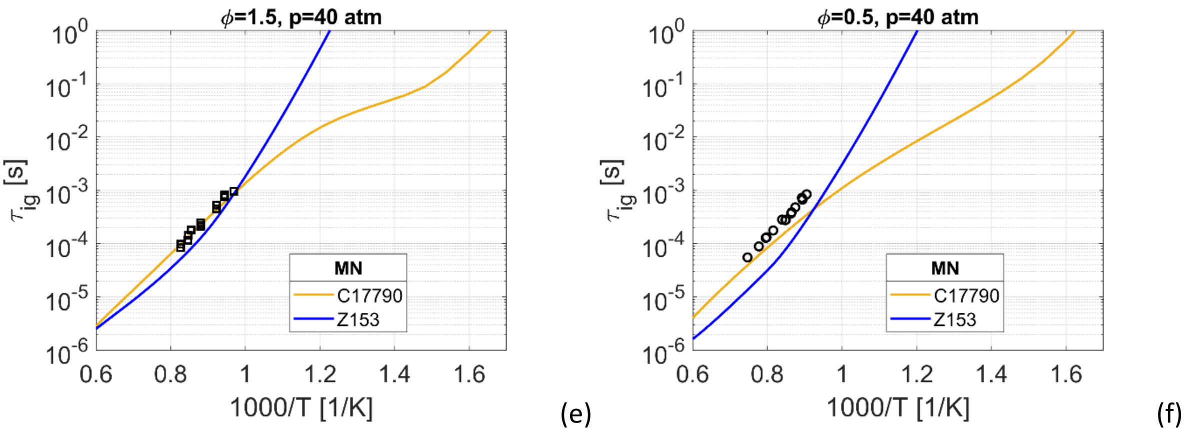

Figure S13. Ignition delay time of MN at p=10 atm and φ=1.5 in (a), p=10 atm and φ=0.5 in (b), p=15 atm and φ=1.0 in (c), p=20 atm and φ=1.0 in (d), p=40 atm and φ=1.5 in (e) and p=40 atm and φ=0.5 in (f). Experimental data: in (a): **o** - (27); in (b): **o** - (27); in (c): **o** - (28), □ - (28), **x** - (29); in (d): **o** - (28); in (e): □ - (27); in (f): **o** - (27).

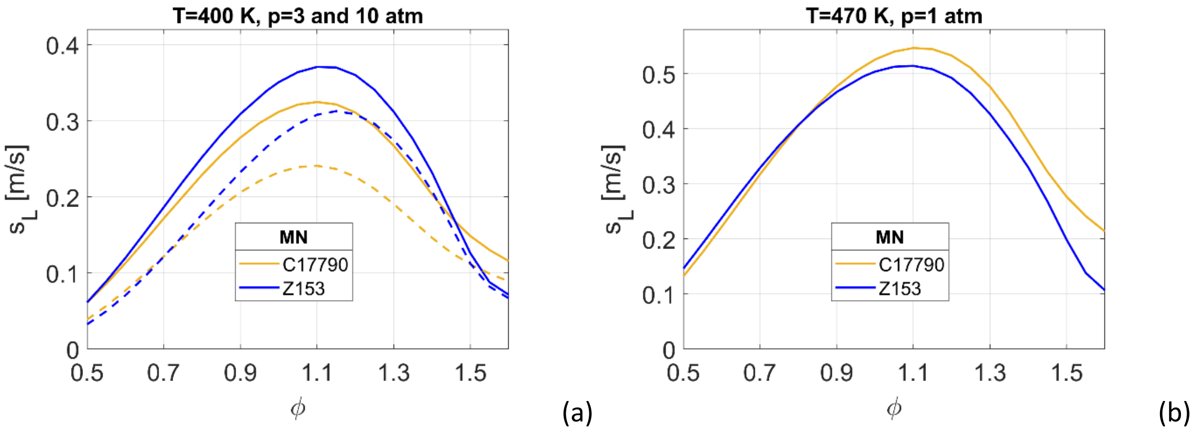

Figure S14. Figure 8. Laminar flame speed for T=400 K and p=3 and 10 atm in (a), and T=470 K and p=1 atm in (b).

Results for n-$C_{12}H_{26}$ / $C_{10}H_7CH_3$ mixtures

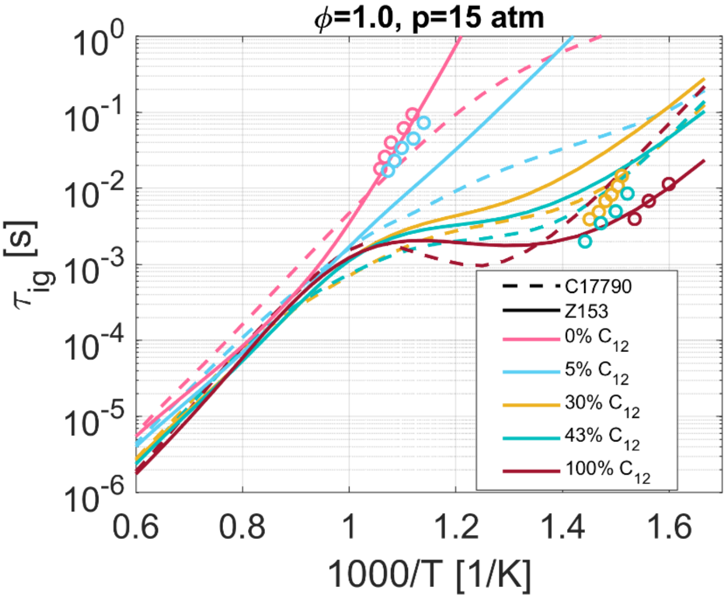

Figure S15. Ignition delay time, p=15 atm and ϕ=1.0, using various n-$C_{12}H_{26}$/MN mixtures. Solid lines for Z153 and dashed for C17790. Experimental data: (29).

References

1. He, X., Donovan, M. T., Zigler, B. T., Palmer, T. R., Walton, S. M., Wooldridge, M. S., & Atreya, A. An experimental and modeling study of iso-octane ignition delay times under homogeneous charge compression ignition conditions. Combust Flame. 2005;142(3):266–75.

2. Shen, H. P. S., Vanderover, J., & Oehlschlaeger, M. A. A shock tube study of iso-octane ignition at elevated pressures: The influence of diluent gases. Combust Flame. 2008;155(4):739–55.

3. Hartmann, M., Gushterova, I., Fikri, M., Schulz, C., Schießl, R., & Maas, U. Auto-ignition of toluene-doped n-heptane and iso-octane/air mixtures: High-pressure shock-tube experiments and kinetics modeling. Combust Flame. 2011;158(1):172–8.

4. Kumar, K., Freeh, J. E., Sung, C. J., & Huang, Y. Laminar flame speeds of preheated iso-octane/O2/N2 and n-heptane/O2/N2 mixtures. J Propuls Power. 2007;23(2):428–36.

5. Shen, H. P. S., Steinberg, J., Vanderover, J., & Oehlschlaeger, M. A. A shock tube study of the ignition of n-heptane, n-decane, n-dodecane, and n-tetradecane at elevated pressures. Energy Fuels. 2009;23(5):2482–9.

6. Pfahl, U., Fieweger, K., & Adomeit, G. Self-ignition of diesel-relevant hydrocarbon-air mixtures under engine conditions. In: Symposium (International) on combustion. Elsevier; 1996. p. 781–9.

7. Kumar, K., & Sung, C. J. Laminar flame speeds and extinction limits of preheated n-decane/O2/N2 and n-dodecane/O2/N2 mixtures. Combust Flame. 2007;151(12):209–24.

8. Hui, X., & Sung, C. J. Laminar flame speeds of transportation-relevant hydrocarbons and jet fuels at elevated temperatures and pressures. Fuel. 2013;109:191–200.

9. Mao, Y., Feng, Y., Wu, Z., Wang, S., Yu, L., Raza, M., ... & Lu, X. The autoignition of iso-dodecane in low to high temperature range: An experimental and modeling study. Combust Flame. 2019;210:222–35.

10. Fang, R., Kukkadapu, G., Wang, M., Wagnon, S. W., Zhang, K., Mehl, M., ... & Sung, C. J. Fuel molecular structure effect on autoignition of highly branched iso-alkanes at low-to-intermediate temperatures: Iso-octane versus iso-dodecane. Combust Flame. 2020;214:152–66.

11. Kumar, K., Sung, C. J., & Hui, X. Laminar flame speeds and extinction limits of conventional and alternative jet fuels. Fuel. 2011;90(3):1004–11.

12. Zhang, C., Li, B., Rao, F., Li, P., & Li, X. A shock tube study of the autoignition characteristics of RP-3 jet fuel. Proc Combust Inst. 2015;35(3):3151–8.

13. Zeng, W., Li, H. X., Chen, B. D., & Ma, H. A. Experimental and kinetic modeling study of ignition characteristics of Chinese RP-3 kerosene. Combust Sci Technol. 2015;187(3):396–409.

14. Flora, G., Balagurunathan, J., Saxena, S., Cain, J. P., Kahandawala, M. S., DeWitt, M. J., ... & Corporan, E. Chemical ignition delay of candidate drop-in replacement jet fuels under fuel-lean conditions: A shock tube study. Fuel. 2017;209:457–72.

15. Vasu, S. S., Davidson, D. F., Hong, Z., Vasudevan, V., & Hanson, R. K. n-Dodecane oxidation at high-pressures: Measurements of ignition delay times and OH concentration time-histories. Proc Combust Inst. 2009;31(1):173–80.

16. Ji, C., Dames, E., Wang, Y. L., Wang, H., & Egolfopoulos, F. N. Propagation and extinction of premixed C5–C12 n-alkane flames. Combust Flame. 2010;157(2):277–87.

17. Chong, C. T., & Hochgreb, S. Measurements of laminar flame speeds of liquid fuels: Jet-A1, diesel, palm methyl esters and blends using particle imaging velocimetry (PIV). Proc Combust Inst. 2011;33(1):979–86.

18. Wang, M., Zhang, K., Kukkadapu, G., Wagnon, S. W., Mehl, M., Pitz, W. J., & Sung, C. J. Autoignition of trans-decalin, a diesel surrogate compound: Rapid compression machine experiments and chemical kinetic modeling. Combust Flame. 2018;194:152–63.

19. Comandini, A., Dubois, T., Abid, S., & Chaumeix, N. Comparative study on cyclohexane and decalin oxidation. Energy Fuels. 2014;28(1):714–24.

20. Oehlschlaeger, M. A., Shen, H. P. S., Frassoldati, A., Pierucci, S., & Ranzi, E. Experimental and kinetic modeling study of the pyrolysis and oxidation of decalin. Energy Fuels. 2009;23(3):1464–72.

21. Zhu, Y., Davidson, D. F., & Hanson, R. K. Pyrolysis and oxidation of decalin at elevated pressures: A shock-tube study. Combust Flame. 2014;161(2):371–83.

22. Yu, L., Wu, Z., Qiu, Y., Qian, Y., Mao, Y., & Lu, X. Ignition delay times of decalin over low-to-intermediate temperature ranges: Rapid compression machine measurement and modeling study. Combust Flame. 2018;196:160–73.

23. Li, B., Zhang, H., & Egolfopoulos, F. N. Laminar flame propagation of atmospheric iso-cetane/air and decalin/air mixtures. Combust Flame. 2014;161(1):154–61.

24. Rao, F., Li, B., Li, P., Zhang, C., & Li, X. Shock-tube study of the ignition of gas-phase 1, 3, 5-trimethylbenzene in air. Energy Fuels. 2014;28(11):6707–13.

25. Diévart, P., Kim, H. H., Won, S. H., Ju, Y., Dryer, F. L., Dooley, S., ... & Oehlschlaeger, M. A. The combustion properties of 1, 3, 5-trimethylbenzene and a kinetic model. Fuel. 2013;109:125–36.

26. Hui, X., Das, A. K., Kumar, K., Sung, C. J., Dooley, S., & Dryer, F. L. Laminar flame speeds and extinction stretch rates of selected aromatic hydrocarbons. Fuel. 2012;97:695–702.

27. Wang, H., Warner, S. J., Oehlschlaeger, M. A., Bounaceur, R., Biet, J., Glaude, P. A., & Battin-Leclerc, F. An experimental and kinetic modeling study of the autoignition of a-methylnaphthalene/air and a-methylnaphthalene/n-decane/air mixtures at elevated pressures. Combust Flame. 2010;157(10):1976–88.

28. Sun, S., Yu, L., Wang, S., Mao, Y., & Lu, X. Experimental and kinetic modeling study on self-ignition of ?-methylnaphthalene in a heated rapid compression machine. Energy Fuels. 2017;31(10):11304–14.

29. Kukkadapu, G., & Sung, C. J. Autoignition study of binary blends of n-dodecane/1-methylnaphthalene and iso-cetane/1-methylnaphthalene. Combust Flame. 2018;189:367–77.
